# Supplementary material for: Upland Yedoma taliks are an unpredicted source of atmospheric methane
Source: Nat Commun. 2024 Jul 18;15:6056. doi: 10.1038/s41467-024-50346-5 (PMC11258132; doi:10.1038/s41467-024-50346-5)
Supplement: Supplementary file 1 — Supplementary Information [file 41467_2024_50346_MOESM1_ESM.pdf]

Supplementary Information

Upland Yedoma taliks are an unpredicted source of atmospheric methane

Walter Anthony[1]*, K. M., P. Anthony[1], N. Hasson[1], C. Edgar[2], O. Sivan[3], E. Eliani-Russak[3], O. Bergman[3,1], B. J. Minsley[4], S. R. James[4], N. J. Pastick[5], A. Kholodov[6], S. Zimov[7], E. Euskirchen[2], M. S. Bret-Harte[2], G. Grosse[8,9], M. Langer[10,8], J. Nitzbon[8]

Affiliations

[1] Water and Environmental Research Center, University Alaska Fairbanks, Fairbanks, Alaska USA
[2] Institute of Arctic Biology, University Alaska Fairbanks, Fairbanks, Alaska USA
[3] Department of Earth and Environmental Sciences, Ben Gurion University of the Negev, Beersheva, Israel
[4]U.S. Geological Survey, Geology, Geophysics, and Geochemistry Science Center, Denver, CO, USA
[5]U.S. Geological Survey, Earth Resources Observation and Science Center, Sioux Falls, SD, USA
[6]Geophysical Research Institute, University Alaska Fairbanks, Fairbanks, Alaska USA
[7]Pacific Geographical Institute of the Russian Academy of Sciences, Northeast Science Station, Cherskiy, Russia
[8]Alfred Wegener Institute Helmholtz Centre for Polar and Marine Research, Potsdam, Germany
[9] University of Potsdam, Institute of Geosciences, Potsdam, Germany
[10] Department of Earth Sciences, Vrije Universiteit Amsterdam, Amsterdam, The Netherlands

*Correspondence and requests for materials should be addressed to K.M.W.A. (kmwalteranthony@alaska.edu)

Any use of trade, firm, or product names is for descriptive purposes only and does not imply endorsement by the U.S. Government.

1. Supplementary Figures

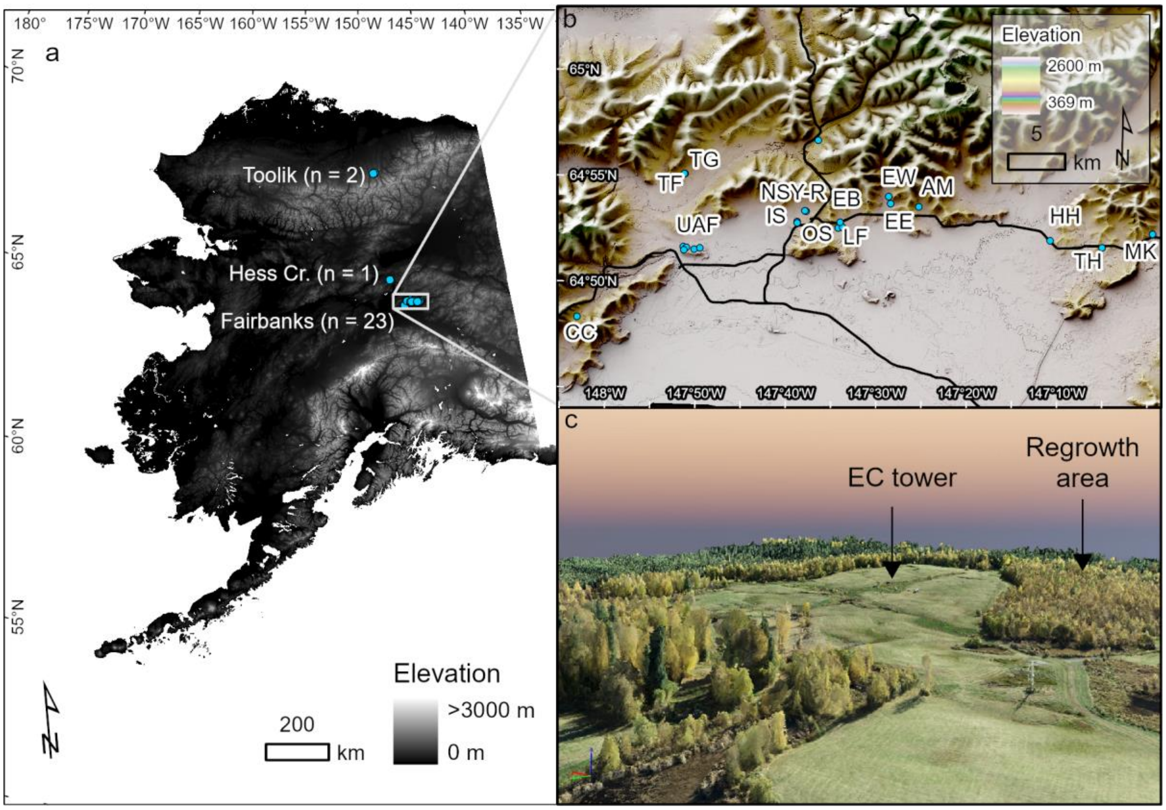

Figure S1. Upland field-study site. a) Alaska-scale digital elevation model[1] and 25 thermokarst-mound study sites (blue dots); b) Fairbanks-area study sites plotted on a hillshade elevation model derived from 2017 USGS 3D Elevation Program LiDAR data[2] with major roads (black lines); c) Aerial photo of North Star Yedoma (NSY) showing location of eddy covariance (EC) tower in an un-mowed (dark green) zone of the graminoid-dominated study field. The image was reconstructed from data collected on a DJI P4 RTK drone equipped with a 20MP camera as part of the NSF-funded Permafrost Grown project (NSF RISE-2126965). In *a* and *b*, the USGS Alaska Science Center and the Alaska Division of Geological and Geophysical Surveys granted permission respectively to use the elevation data, which are in the public domain. Metadata for the 25 Alaska thermokarst-mound study sites are provided in Table S1. Coordinates for adjacent control sites lacking thermokarst mounds (not shown here) are also provided in Table S1.

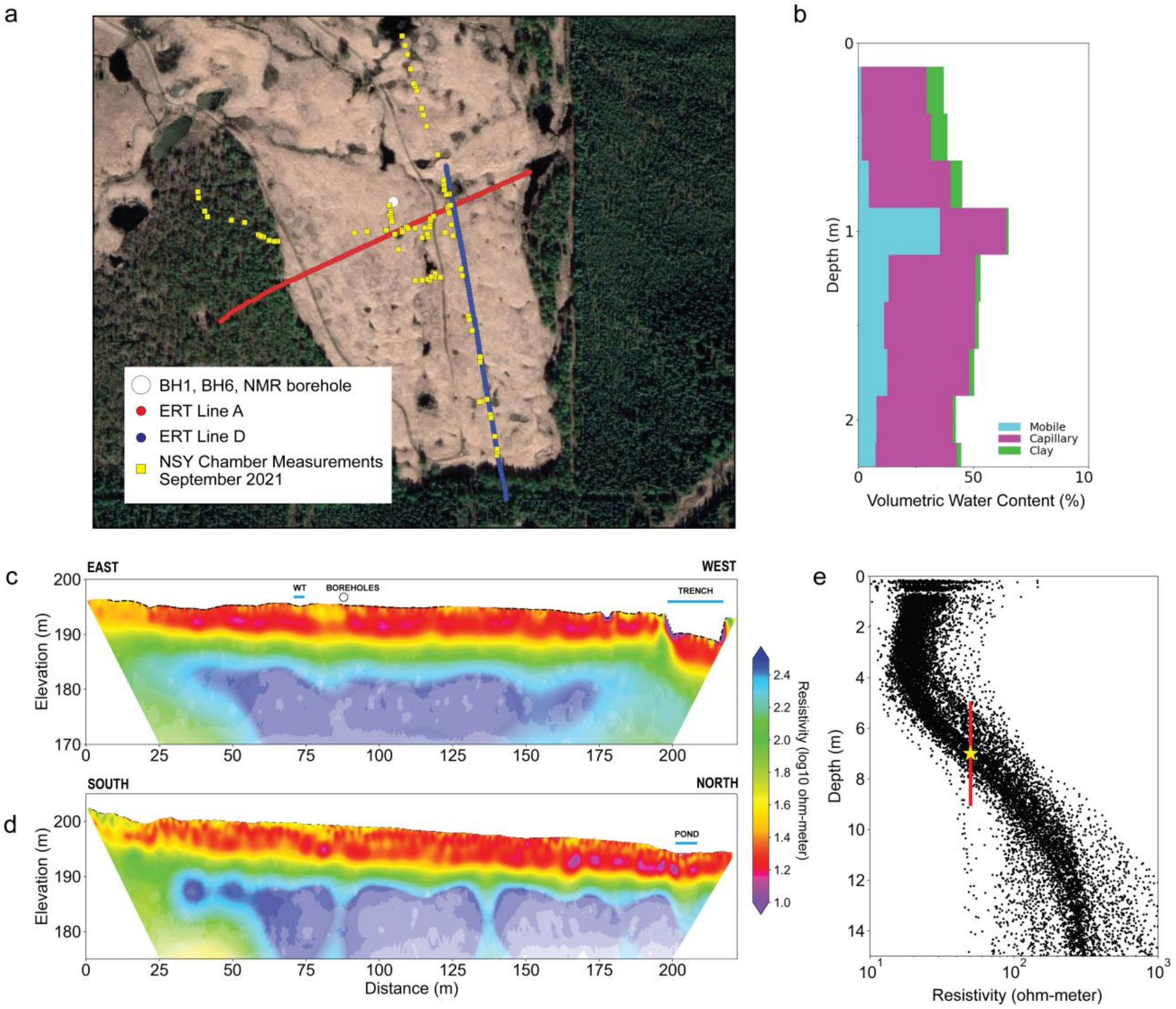

Figure S2. Water content and permafrost distribution based on geophysics and borehole drilling at NSY. a) Locations of electrical resistivity tomography (ERT) transects and borehole nuclear magnetic resonance (NMR), located within ~1-m of soil core boreholes BH1 and BH6; b) Soil water content inferred from NMR; c) North Star A ERT transect with key features annotated (Water track = WT); d) North Star D ERT transect; e) all resistivity values as a function of depth for the upper 15 m. Resistivity observed on Line A nearest to BH6 at the depth to the top of permafrost (6.8 m) is approximately 50 ohm-m (yellow star). Using this resistivity threshold suggests a range in depth to permafrost between ~5-9 m (red line) across all locations. Details of geophysical measurements are provided in the Methods section and Minsley et al.[3].

=

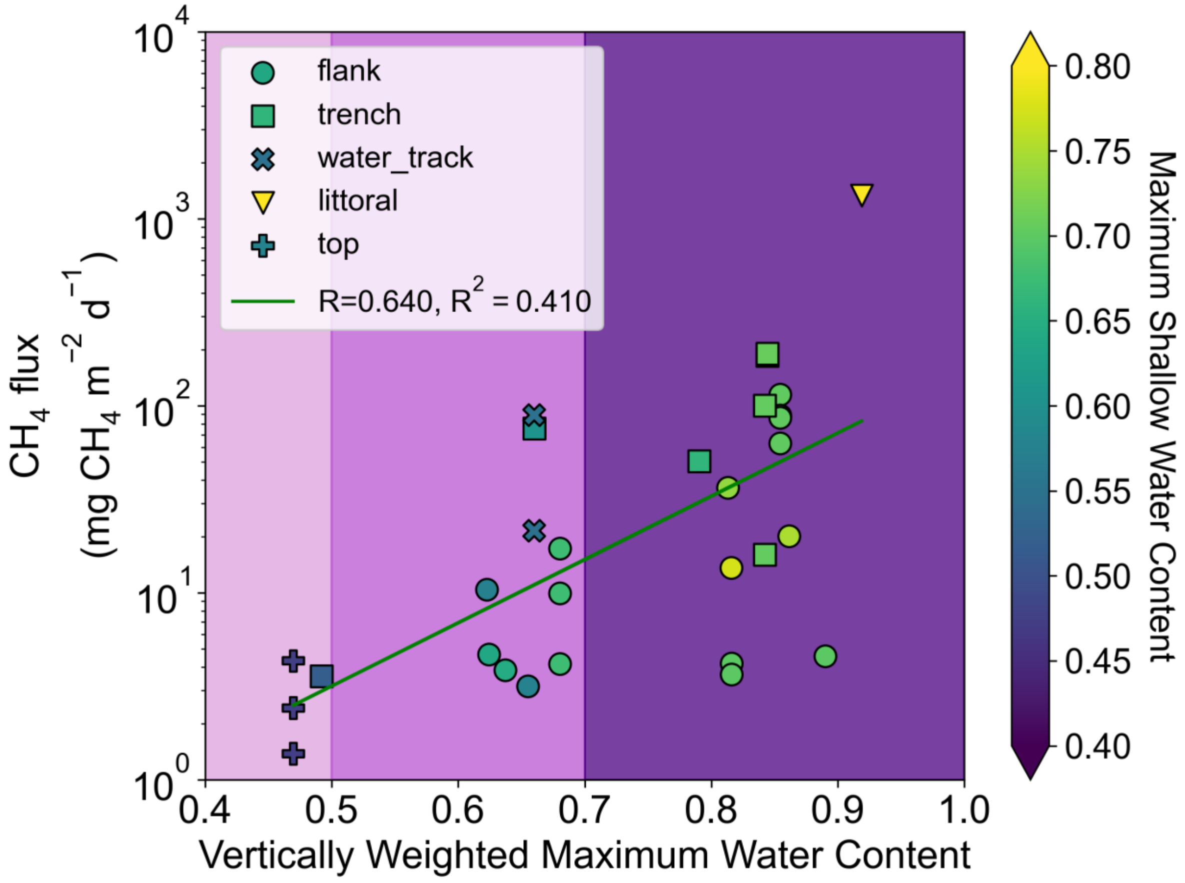

Figure S3. Empirical geophysics-methane flux relationship in September 2021. Methane (CH_4) flux measurements (4 zero and 2 negative fluxes excluded) are displayed against scaled water content estimated from ERT transect locations within 2 m of chamber observations. Maximum water content in the uppermost 3 m is a primary driver in the relationship to observed fluxes. The relationship is classified into three groups that correlate with low (light pink), moderate (purple), and high (dark purple) fluxes that are mapped back onto all ERT transect locations in Fig. 4; the zero and negative fluxes are also shown in Fig. 4.

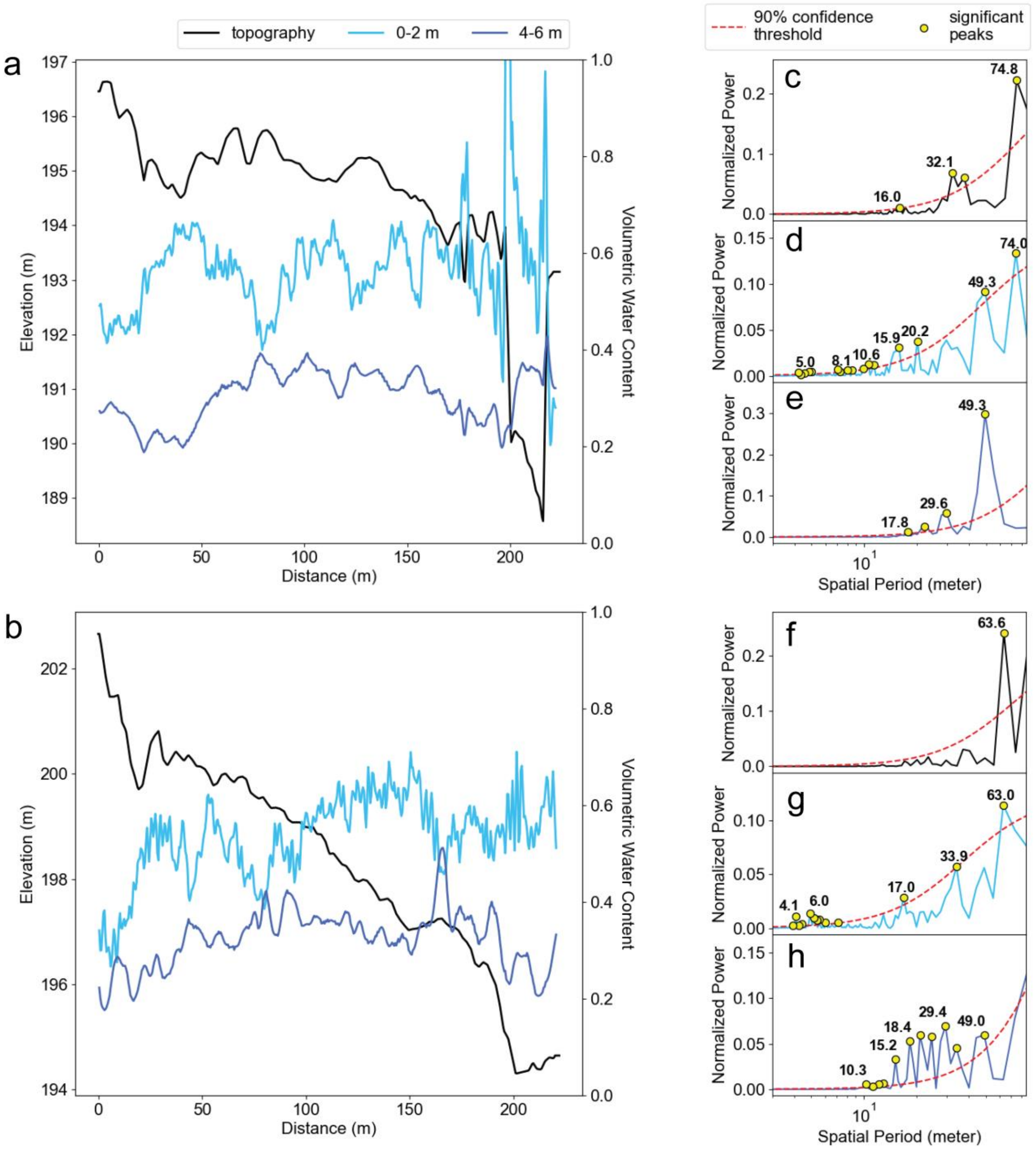

Figure S4. Spatial periodicity of geophysically-derived water content. Ground surface topography in comparison to water content at shallow and deep depth intervals along the a) Line A and b) Line D ERT profiles. The fast-fourier transform (FFT) of c,f) ground surface topography and ERT water content signals within the d,g) 0-2 m, and e,h) 4-6 m depth intervals for Lines A and D, respectively, show statistically significant spectral peaks with >90% confidence.

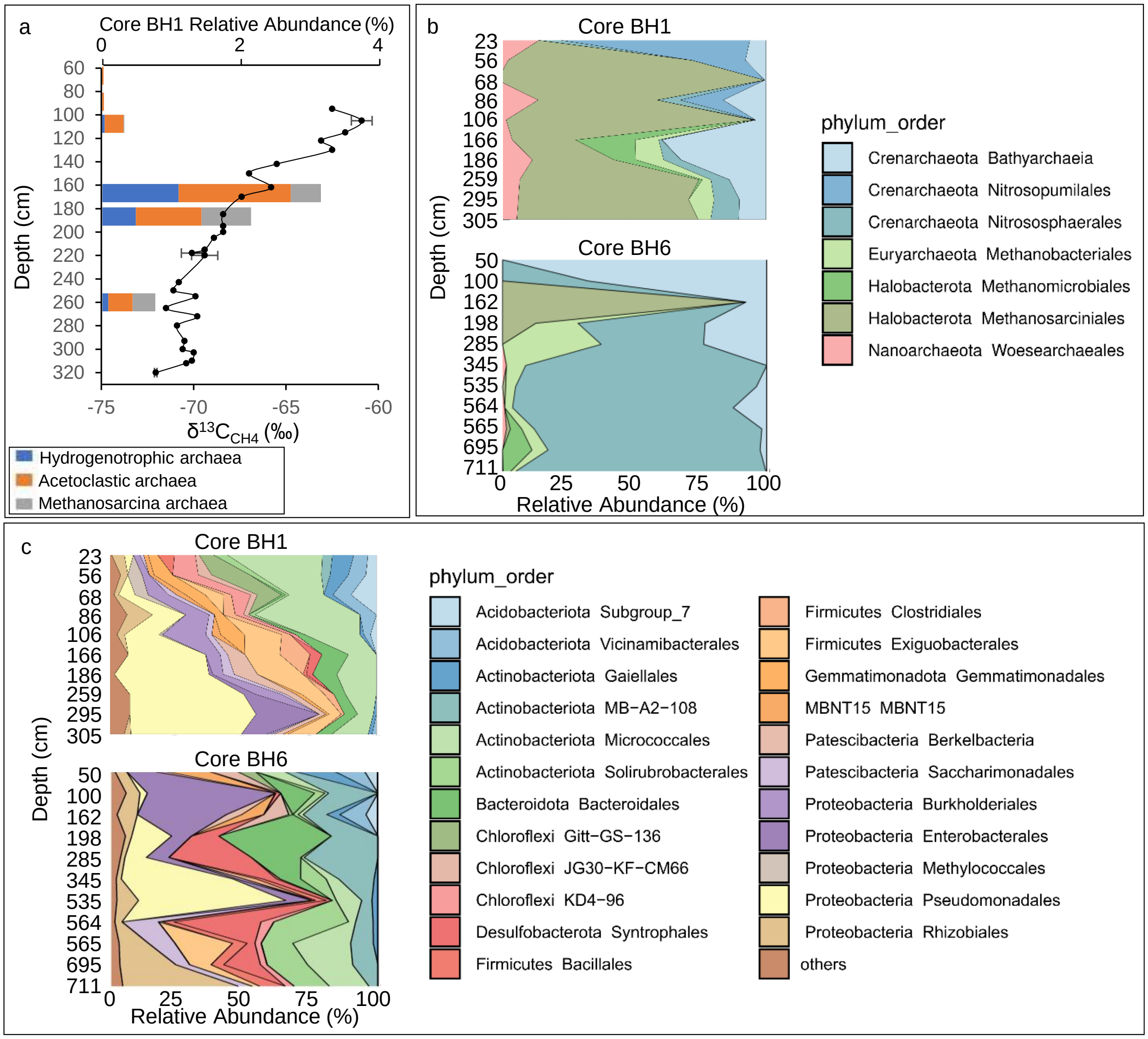

Figure S5. Soil core microbes. a) The $\delta^{13}C_{CH4}$ (solid curve) values and the relative abundance of methanogens [hydrogenotrophic archaea (blue), acetoclastic archaea (orange), and methanosarcina archaea (grey), including both hydrogenotrophic and acetoclastic pathways] in the BH1 core. Error bars for $\delta^{13}C_{CH4}$ are the SD of n=2 technical replicates. b-c) Relative abundance presenting the most abundant classified Amplicon Sequence Variants (ASVs) at the phylum and order level classification for archaea (b) and bacteria (c) in NSY soil depth profiles. The relative abundance of bacterial ASVs significantly higher than archaea (96.3% and 3.7%): 322 bacteria orders, 9 archaea orders. The threshold used for ASVs relative abundance in panels b and c was >1%.

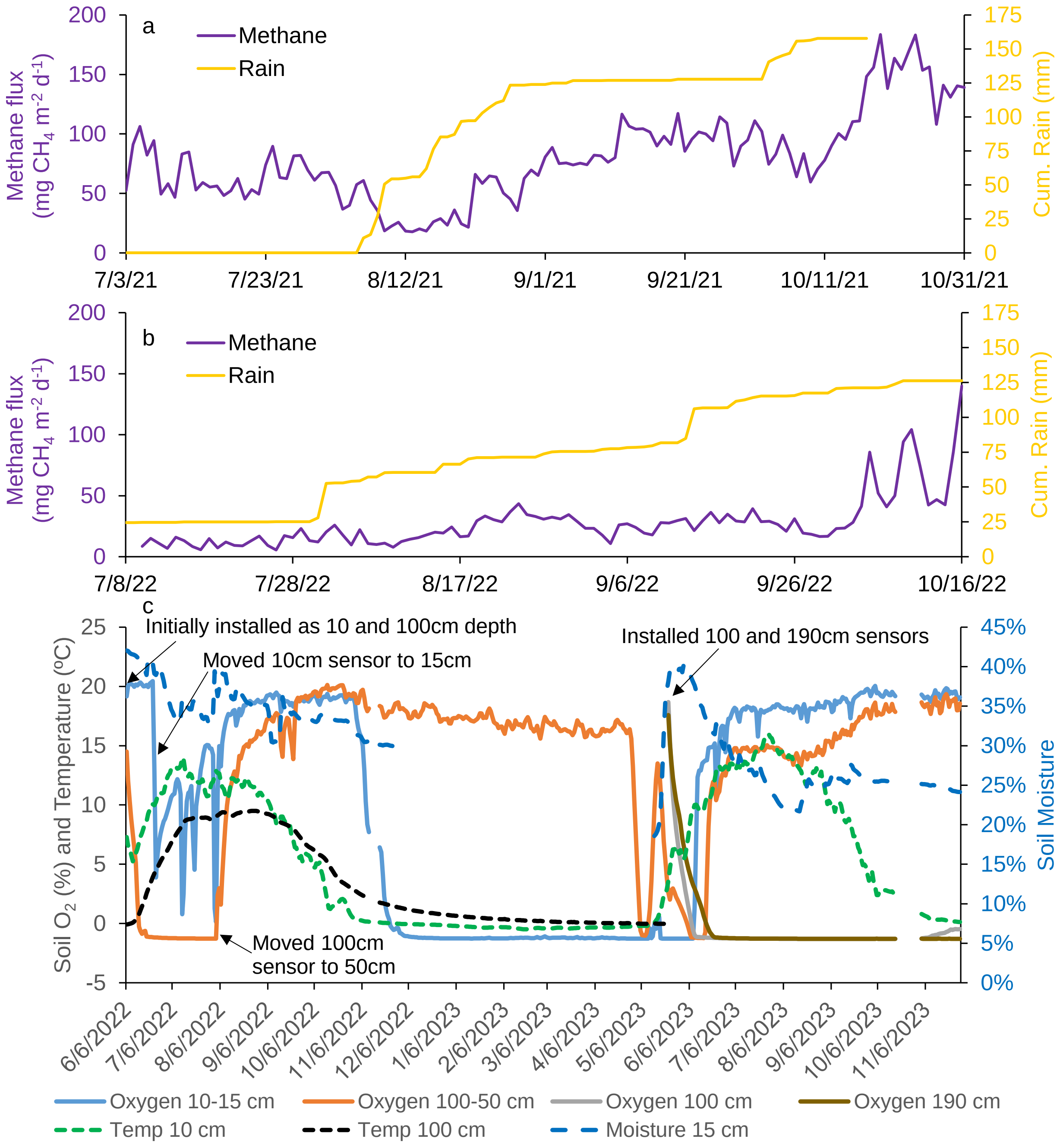

Figure S6. Methane (CH_4) flux, rain, and soil oxygen, temperature, and moisture measured at the eddy covariance tower site at NSY. a) Summer 2021 methane and cumulative rainfall. b) Summer 2022 methane and cumulative rainfall. c) Soil oxygen, temperature, and moisture (VWC).

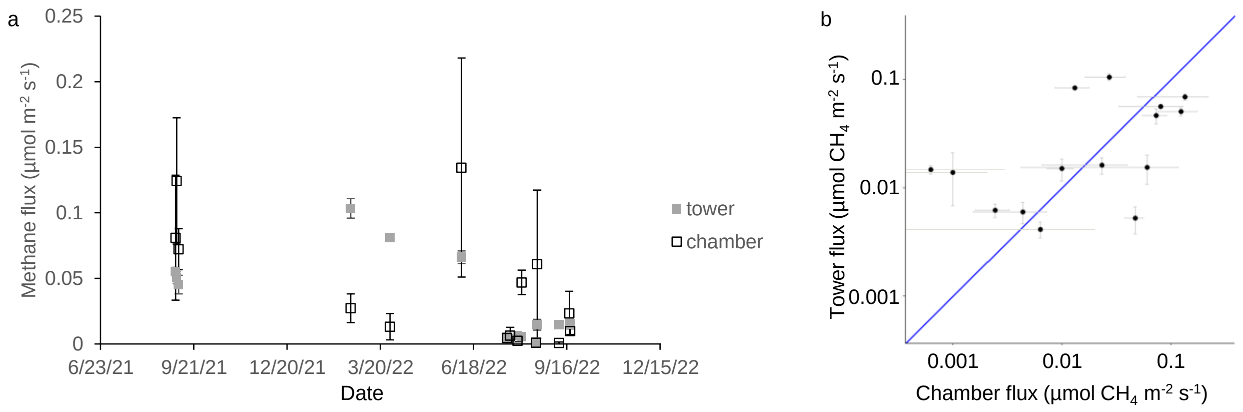

Figure S7. Comparison of NSY methane fluxes derived by eddy covariance (tower) and chambers. a) Chamber and tower flux measurements averaged over the time period of plot-level measurements are shown by date. b) Scatterplot of chamber and tower fluxes shows an even distribution of means above and below the 1:1 line (blue line), indicating an approximate 1:1 relationship between chamber and tower data. Uncertainties are represented by SEM. Error bars are the SEM of biological replicates for the following samples sizes (chamber,tower) by date: 9/3/21 (22/3), 9/4/21 (69,12), 9/6/21 (11,4), 2/19/22 (33,9), 3/29/22 (13,3), 6/6/22 (53,16), 7/20/22 (25,7), 7/23/22 (15,4), 7/30/22 (34,4), 8/3/22 (15,4), 8/17/22 (40,9), 8/18/22 (14,9), 9/8/22 (27,4), 9/18/22 (27,5), 9/19/22 (69,12).

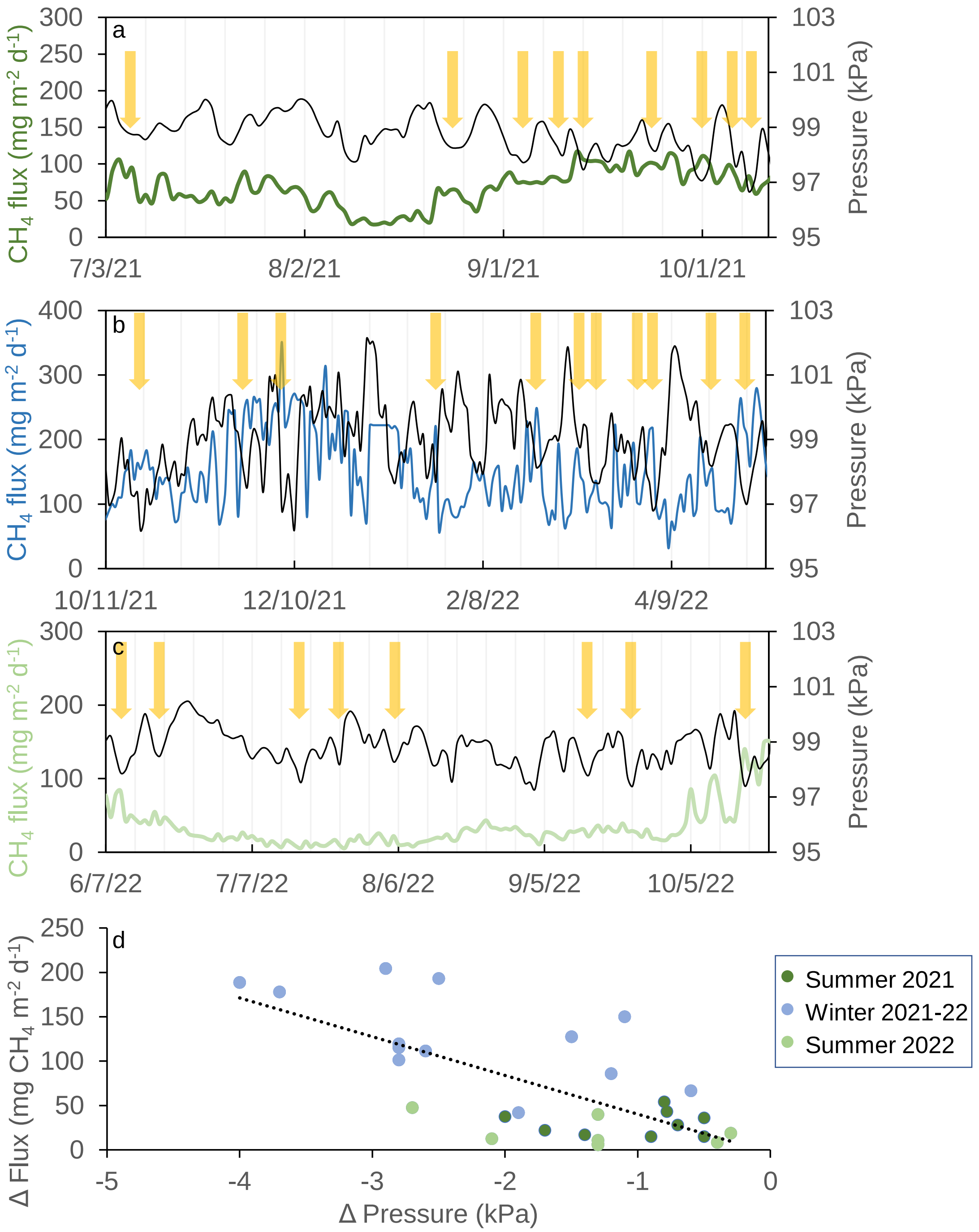

Figure S8. Barometric pressure and methane dynamics at NSY. Changes in selected troughs of barometric pressure (yellow arrows) with synchronous increases in methane flux were recorded for summer 2021 (a), winter 2021-22 (b) and summer 2022 (c). d) The inverse relationship between decreases in atmospheric pressure and rise in methane flux for the selected periods was $y = -43.6x - 3.3$, (r^2=.47, $F(1,28)=26.3$, $p<.001$).

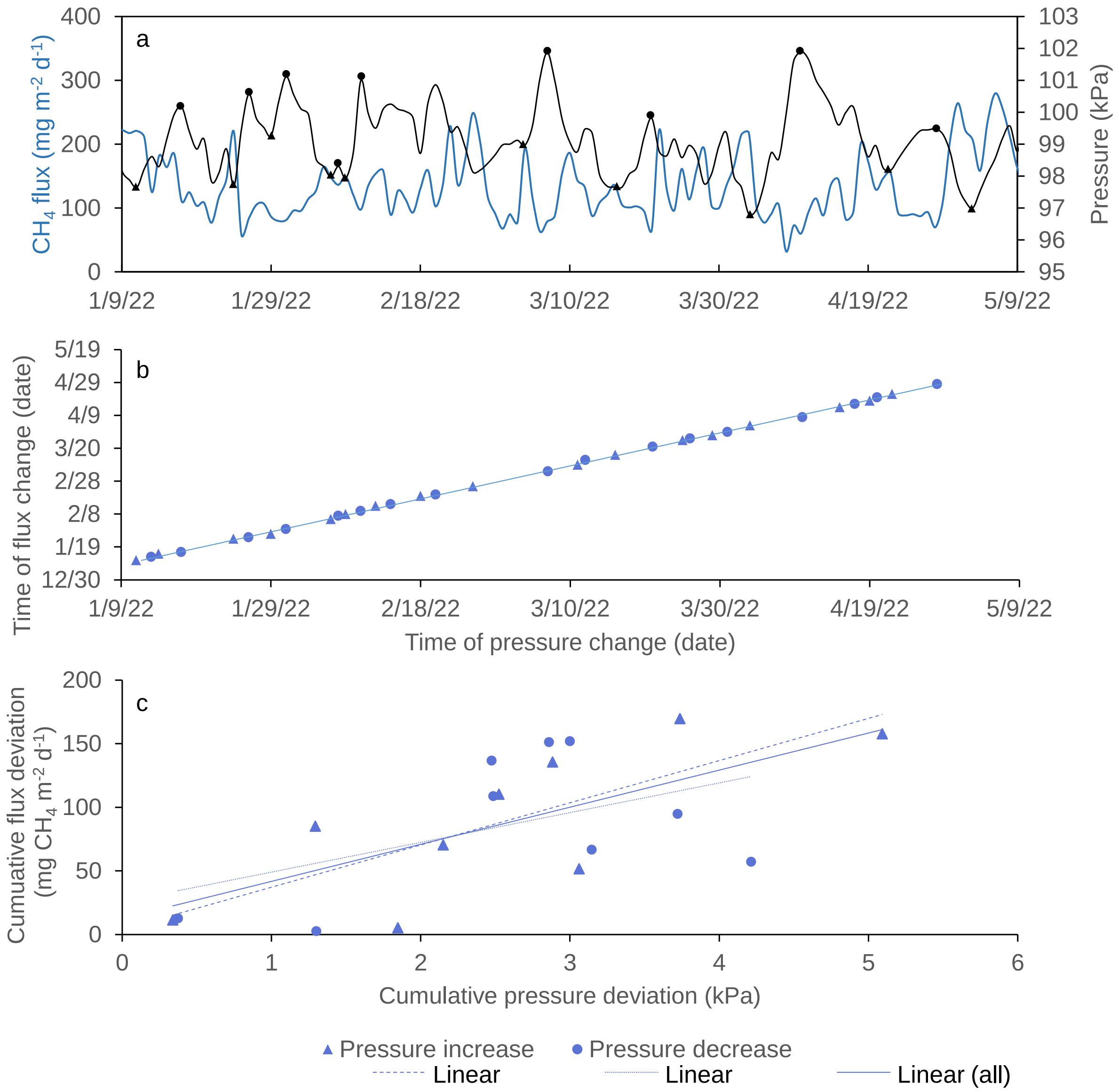

Figure S9. Time-integrated relationship between barometric pressure and methane flux at NSY. a) Barometric pressure and methane observations with pressure inflection points indicating the start of data segments of cumulative pressure increase (triangles) or decrease (circles) during winter 2022. b) The correlation between the time of pressure change and time of flux change for pressure change events (y=1.0x+168.0, r^2=.99). c) Cumulative pressure deviation plotted against cumulative methane flux deviation during time-integrated intervals of pressure change for segments indicated in panel a. The correlation between all (positive and negative) cumulative deviations in *c* was y = 29.0x + 12.7 (r^2=.41, F(1,16)=11.2, p=.004).

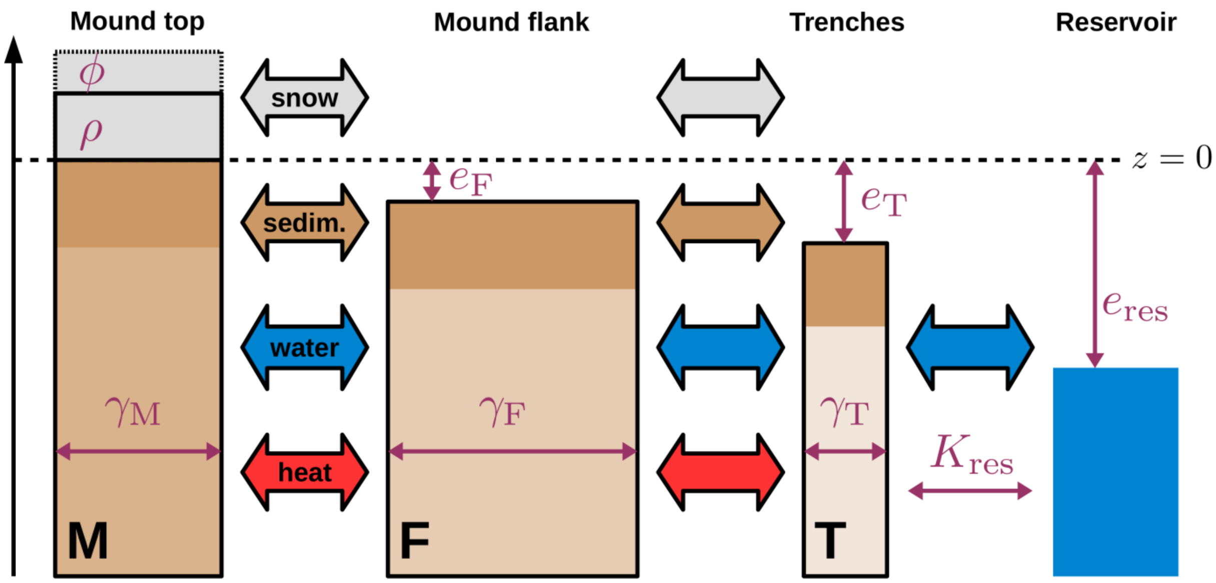

Figure S10. Schematic of the tile-based model setup and key model parameters. The upland Yedoma landscape is represented via three tiles corresponding to mound tops (M), mound flanks (F), and trenches (T) which differ primarily in their excess ice content. Heat, water, sediment, and snow are transported laterally among the tiles, and excess water from the trenches can drain into an external reservoir. See Table S5 for an overview of the key model parameters and Table S6 for the ground stratigraphies.

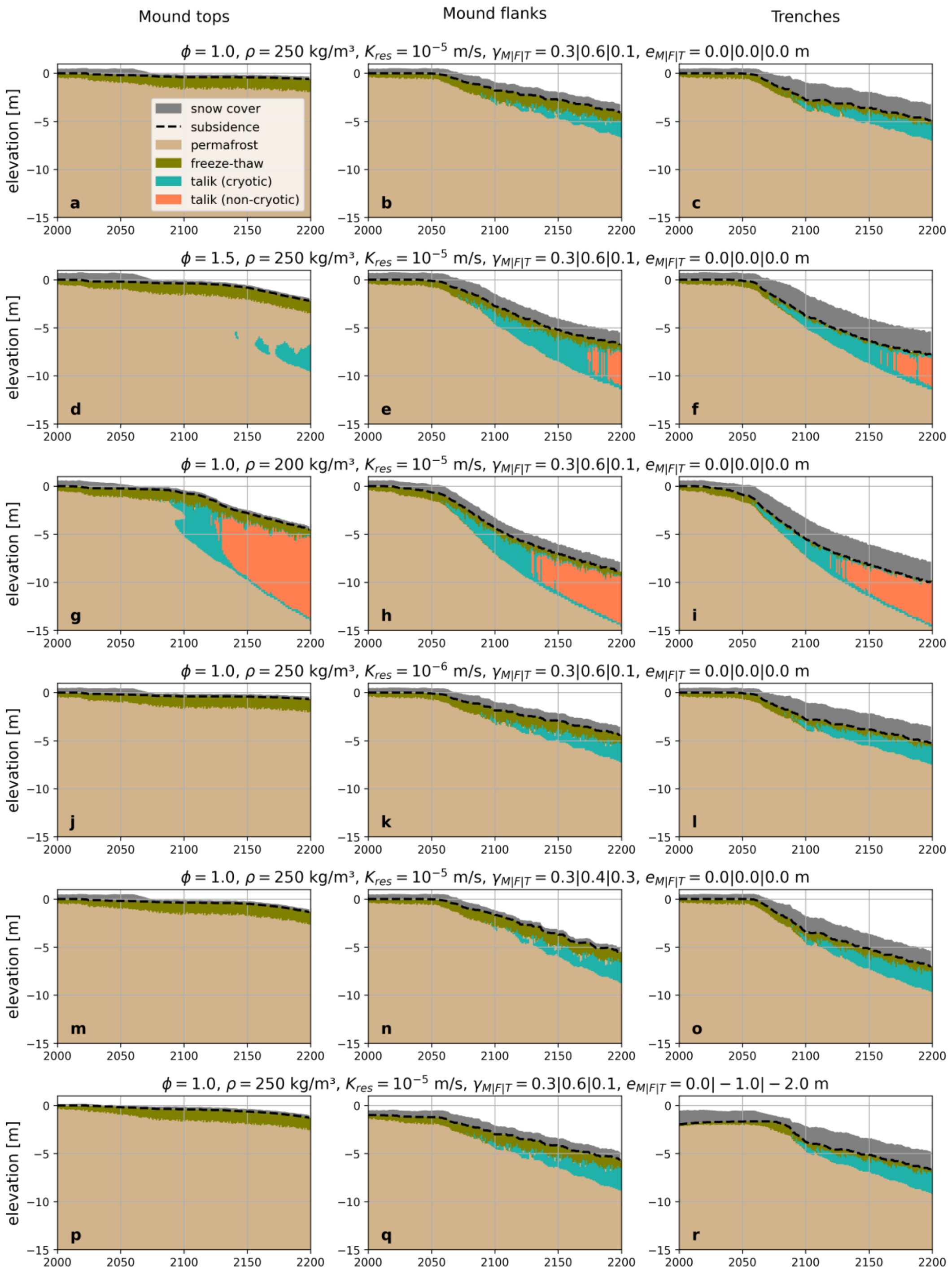

Figure S11. Simulated thermokarst-mound and talik formation for the Lena river delta in northeastern Siberia under RCP8.5 under different snow conditions. Thaw-subsidence (dashed line), maximum snow heights (gray area), and evolution of the hydorthermal state (coloured areas) are shown for mound tops (a,d,g,j,m,p), mound flanks (b,e,h,k,n,q), and trenches (c,f,i,l,o,r). The rows correspond to different simulations under variation of model parameters as indicated in Table S5.

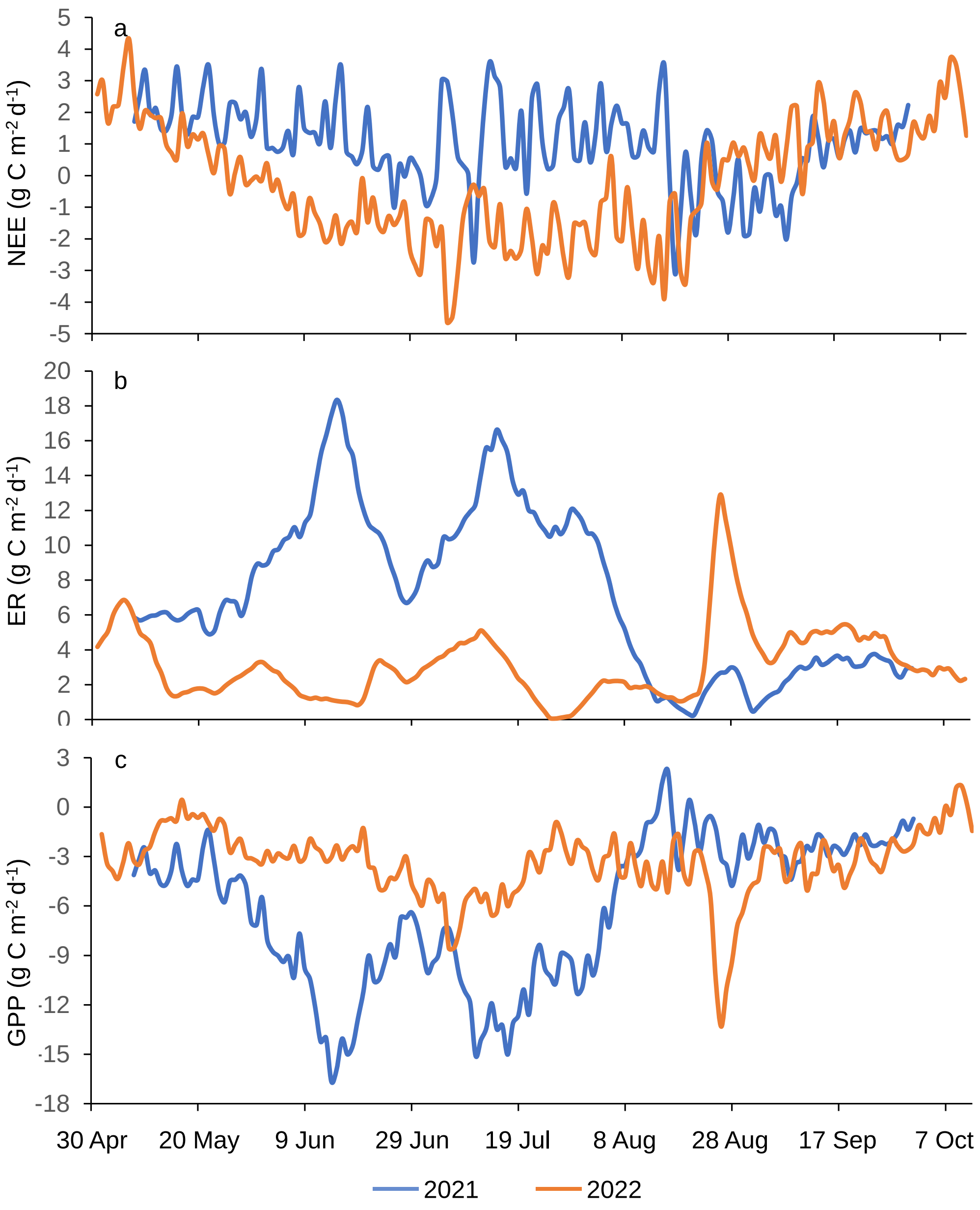

Figure S12. Daily net ecosystem exchange (NEE, panel a), ecosystem respiration (ER, panel b), and gross primary productivity (GPP, panel c) during the snow-free period at the NSY eddy covariance tower. NEE is the difference between gross CO_2 assimilation (GPP), where GPP < 0 because CO_2 uptake is denoted as a negative value, and ER, a positive value.

2. Supplementary Tables

Table S1a. Study site metadata. Metadata including site-name abbreviations used in Fig. S1, location, year of disturbance if known, and present-day dominant vegetation.

No.	Name	Abbreviation	Latitude	Longitude	Disturbance	Vegetation
			Thermokarst Mound Sites			
1	Heritage Hills	HH	64.8749	-147.1845	<1986	Decidious forest
2	Isabella	IS	64.8844	-147.6708	1967-1985	Graminoid
3	Fox CRREL Uplands	FOX	64.9498	-147.6177	< 1995	Decidious forest
4	Trice Grasto Spruce Forest	TF	64.9210	-147.8653	< 1967	Coniferous forest
5	North Star Yedoma	NSY	64.8939	-147.6373	1976-1978	Graminoid
6	E6 (Toolik)	E6	68.6461	-149.4450	-	Tundra
7	Lindig Farm East	LFa	64.8830	-147.5711	<1967	Graminoid
8	Trice Grasto Graminoid	TG	64.9207	-147.8639	< 1967	Graminoid
9	Amanita	AA	64.8995	-147.4281	<1985	Coniferous forest
10	NSY Regrowth	NSY-R	64.8940	-147.6399	1976-1978	Decidious forest
11	Thoreau	TH	64.8700	-147.0881	<1978	Decidious forest
12	Hess Creek	HC	65.5620	-148.9142	1974-1977	Coniferous forest
13	Esro West	EW	64.9070	-147.4844	< 1985	Decidious forest
14	Lindig Farm Field	LFc	64.8819	-147.5690	1949-1967	Graminoid
15	Eastview-Bennett	EB	64.8858	-147.5745	1949-1967	Decidious forest
16	CrippleCreek	CC	64.8057	-148.0564	-	Coniferous forest
17	Lindig Farm Bear Trail	LFb	64.8813	-147.5776	<1985	Coniferous forest
18	UAF Potato Field	UAFp	64.8633	-147.8630	1908	Graminoid
19	Esro East	EE	64.9014	-147.4807	< 1985	Decidious forest
20	UAF Murie	UAFm	64.8613	-147.8433	<1967	Coniferous forest
21	Old Steese	OS	64.8853	-147.6530	<1949	Decidious forest
22	NE2 (Toolik)	NE2	68.6422	-149.5829	-	Tundra
23	McKee Farm	MK	64.8812	-146.9943	<1976	Graminoid
24	UAF Groomers	UAFg	64.8625	-147.8319	<1949	Coniferous forest
25	UAF Bicycle Bumps	UAFb	64.8630	-147.8578	1908	Decidious forest
26	UAF Midnight Sun Loop	UAFs	64.8608	-147.8624	<1949	Decidious forest
			Control Sites			
		AA control	64.8993	-147.4291		Coniferous forest
		CR control	64.8109	-148.0071		Deciduous forest
		EE control	64.8974	-147.4783		Deciduous forest
		FOX control	64.9498	-147.6177		Coniferous forest
		HC control	65.5684	148.9238		Tundra
		IS-a control	64.8840	-147.6683		Coniferous forest
		IS-b control	64.8852	-147.6730		Coniferous forest
		LF-a control	64.8825	-147.5650		Coniferous forest
		LF-b control	64.8804	-147.5795		Mixed forest-tundra
		NE2 control	68.6422	-149.5829		Tundra
		NSY control	64.8933	-147.6344		Coniferous forest
		NSY-R control	64.8923	-147.6370		Coniferous forest
		OS control	64.8856	-147.6530		Deciduous forest
		TG control	64.9204	-147.8661		Coniferous forest
		TH control	64.8735	-147.0919		Coniferous forest
		UAF control	64.8604	-147.8567		Mixed forest

Table S1b. Chamber-based methane and carbon dioxide fluxes and soil moisture measured in upland thermokarst mounds and control sites lacking mounds on all dates. Since chambers were semi-shrouded (see Methods), CO_2 fluxes are an imperfect measure of soil respiration. Among 26 thermokarst-mound study sites, two-way ANOVA on rank-transformed data revealed no interaction between site and season on methane flux ($F(3,1112)=2.0$, $p=.11$), but simple main effect of site was significant ($p<.001$). Site-specific methane emissions are listed in mean rank order from highest to lowest for thermokarst-mounds. Two-way ANOVA revealed no significant interaction between study sites and type (thermokarst-mound vs. control) on plot-scale chamber methane fluxes ($F(10,609)=1.32$, $p=.21$), but simple main effects showed that emissions from thermokarst mounds were higher than from the adjacent controls ($p=.00001$).

		Methane Flux (mg CH_4 m^{-2} d^{-1})					Carbon Dioxide Flux (g CO_2 m^{-2} d^{-1})					Soil Moisture (%)				
Site	Field Date	Mean ± SEM	n	median	min	max	Mean ± SEM	n	median	min	max	Mean ± SEM	n	median	min	max
							Thermokarst Mound Sites									
HH	8/15/2022	662.6 ± 640.5	10	10.7	-2.0	6426.6	18.7 ± 4.6	10	19.2	0.0	41.0	47.1 ± 4.9	10	48.8	12.1	67.9
IS	8/11/22-2/27/23	327.8 ± 255.7	62	2.7	-2.2	15648.1	27.7 ± 16.6	63	11.1	-4.8	1052.7	33.6 ± 1.6	35	35.7	5.3	44.3
Fox	5/27/2022	143.1 ± 73.4	6	91.3	7.8	502.7	3.9 ± 1.0	6	4.1	-0.1	7.0					
TF	5/28/2022-2/28/23	110.2 ± 60.7	18	7.3	2.5	965.0	4.8 ± 1.3	13	3.1	2.0	20.0	12.7 ± 5.0	7	8.8	3.9	41.8
NSY	3/18/20-3/11/23	110.2 ± 24.1	665	7.6	-2.5	12790.1	7.6 ± 0.3	644	6.1	-43.8	55.2	43.9 ± 0.4	306	43.4	24.0	89.3
E6	9/6/2022	72.6 ± 40.7	4	49.7	1.7	189.4	3.6 ± 2.2	4	4.0	-1.7	8.0	26.3 ± 10.6	4	17.0	13.5	57.8
LFa	8/12/22-2/28/23	70.2 ± 34.8	38	7.2	-2.9	1263.4	13.5 ± 2.0	38	14.2	-11.8	32.7	37.0 ± 2.0	20	38.6	17.5	49.5
TG	5/28/2022-2/28/23	69.8 ± 20.9	43	12.2	0.9	533.7	7.4 ± 1.0	46	7.3	-14.9	23.2	34.5 ± 3.1	13	39.3	12.1	46.6
AA	6/7/2022	46.1 ± 22.7	3	52.1	4.1	82.0	17.7 ± 1.0	3	17.6	16.0	19.4	25.6 ± 3.7	2	25.6	21.9	29.2
NSY-R	3/18/20-8/17/22	34.2 ± 13.4	58	3.9	-2.7	574.9	19.0 ± 1.9	61	15.9	0.0	78.3	36.2 ± 1.4	45	35.5	19.3	75.2
TH	8/13/2022	32.8 ± 26.8	27	-1.2	-4.3	721.5	42.1 ± 3.9	27	35.5	12.9	105.2	14.4 ± 1.5	27	15.0	0.2	29.8
HC	9/7/2022	32.3 14.5	7	19.7	-1.0	109.9	10.2 2.3	6	9.7	3.3	18.0	31.9 ± 7.5	4	34.3	13.3	45.8
EW	3/28/2021	30.7 ± 30.7	5	0.0	0.0	153.3	0.0 ± 0.0	5	0.0	0.0	0.0					
LFc	8/13/2022	11.1 ± 7.8	2	11.1	0.0	22.1	15.7 ± 4.0	2	15.7	10.1	21.4	33.6	1			
EB	8/8-8/28/22	7.5 ± 3.8	37	0.0	-3.9	104.8	17.5 ± 1.8	37	16.3	1.9	48.3	36.6 ± 2.6	37	35.4	7.2	66.5
CC	2/21/2022	5.4	1	5.4			1.7	1	1.7							
LFb	8/13/2022	5.0 ± 4.0	5	2.4	-1.0	20.9	10.1 ± 3.7	5	13.6	-3.4	17.6	36.8 ± 3.8	5	40.3	21.6	41.9
UAFp	6/29/22-3/10/23	5.0 ± 3.1	58	0.0	-4.2	161.4	15.8 ± 1.4	64	15.4	-3.6	39.5	37.2 ± 2.0	18	37.4	20.7	48.2
EE	10/1/2020	2.7 ± 1.5	18	0.0	-2.1	23.1	13.1 ± 2.2	18	12.3	2.3	35.7					
UAFm	3/26-3/27/2020	1.9 ± 1.7	18	0.0	0.0	30.2	28.3 ± 19.7	18	0.0	0.0	347.6					
OS	8/10/2022	1.2 ± 1.3	17	0.0	-2.5	21.7	17.4 ± 2.3	17	14.6	0.0	36.4	33.9 ± 6.8	17	27.1	0.0	91.5
NE2	9/4/2022	0.6 ± 0.7	4	1.2	-1.4	1.6	0.9 ± 2.2	4	2.6	-5.4	4.1	43.3 ± 5.5	2	43.3	37.8	48.8
MK	8/13/2022	0.0 ± 3.2	9	-2.1	-14.1	22.0	213.0 ± 172.5	9	35.9	4.2	1590.3	41.9 ± 1.8	2	41.9	40.1	43.7
UAFg	3/26/20	0.0 ± 0.0	16	0.0	0.0	0.0	3.3 ± 2.4	16	0.8	0.0	39.5					
UAFb	3/26/20-8/27/22	-1.4 ± 0.3	40	-0.9	-6.5	0.4	22.4 ± 3.9	40	22.2	0.0	72.6	8.2 ± 1.3	40	12.1	0.1	20.2
UAFs	8/14-8/27/22	-4.2 ± 0.5	8	-3.8	-6.7	-2.6	54.8 ± 10.3	8	52.7	19.1	108.6	5.6 ± 1.3	8	4.9	0.4	13.0
							Control Sites									
AA control	6/7/22	0.0 ± 0.0	2	0.0	0.0	0.0	4.0 ± 1.2	2	4.0	2.8	5.2	12.2 ± 4.6	2	12.2	7.6	16.7
CR control	8/27/22	-4.7 ± 0.5	3	-4.4	-5.8	-3.9	24.1 ± 2.6	3	26.5	18.8	27.0	7.3 ± 0.8	3	7.7	5.8	8.4
EE control	9/15/23	-1.0 ± 1.0	5	0.0	-5.1	0.0	15.4 ± 2.3	5	13.6	11.7	24.4	13.6 ± 2.3	4	15.3	6.8	16.9
Fox control	5/27/22	-0.5 ± 0.3	5	0.0	-1.4	0.0	11.6 ± 1.8	5	13.3	5.8	15.3					
HC control	10/14/22	-0.1 ± 0.1	18	0.0	-0.7	0.0	1.8 ± 0.2	16	1.8	0.6	2.6					
ISa control	9/19/23	0.0 ± 0.0	3	0.0	0.0	0.0	6.1 ± 1.2	3	7.0	3.8	7.6	0.0 ± 0.0	2	0.0	0.0	0.0
ISb control	9/19/23	0.0 ± 0.0	7	0.0	0.0	0.0	9.1 ± 3.1	5	4.3	4.1	17.8	2.5	1			
LFa control	9/13/23	-1.0 ± 1.0	6	0.0	-5.7	0.0	16.0 ± 3.5	6	14.4	8.6	31.5	15.0 ± 4.1	3	15.1	7.9	22.1
LFb control	8/13/22	-3.5 ± 1.0	3	-4.0	-4.9	-1.5	9.9 ± 2.1	3	10.4	6.1	13.3	29.7	1	29.7		
NE2 control	9/4/22	-1.7	1	-1.7			1.5	1				37.8	1			
NSY control	9/8/23	0.0 ± 0.0	4	0.0	0.0	0.0	7.3 ± 3.3	2	7.3	3.9	10.6					
NSY-R control	3/18/2020-9/8/23	-0.7 ± 0.7	6	0.0	-3.9	0.0	20.5 ± 5.6	6	18.0	6.1	42.3	6.2 ± 3.3	3	7.2	0.0	11.3
OS control	8/10/22	0.0 ± 0.0	3	0.0	0.0	0.0	14.5 ± 3.7	3	13.6	8.5	21.2	24.7 ± 11.6	3	24.9	4.6	44.7
TG control	9/14/23	0.0 ± 0.0	6	0.0	0.0	0.0	11.4 ± 1.2	6	10.5	8.7	16.5	19.2 ± 2.9	2	19.2	16.3	22.0
TH control	9/13/23	-1.9 ± 1.2	5	0.0	-5.5	0.0	21.2 ± 5.3	5	14.0	11.9	39.1					
UAF control	8/27/22	-1.3	1	-1.3			22.4	1				2.4	1			

Table S2. Names and locations of select thermokarst-mound landscapes in the pan-Arctic identified in 2020 through 2022 ESRI satellite imagery* and/or field work**.

ID	Location	Region	Lat	Long	Field Truth**
1	Adytsha River	NE Siberia	67.626281	135.453278	
2	Alazeya River	NE Siberia	69.642566	155.014529	
3	Alazeya River	NE Siberia	69.921785	154.943016	
4	Alazeya River	NE Siberia	70.744155	154.011905	
5	Anyui River	NE Siberia, Kolyma	68.308370	161.739288	
6	Anyui River, Inuiy	NE Siberia	68.242528	161.894438	yes
7	Banks Island	NW Canada	72.675739	-119.191130	
8	Belkovsky Island	New Siberian Islands	75.622802	135.789434	
9	Bolshoy Begichev Island	NE Siberia	74.385934	112.277124	
10	Bolshoy Lyakhovsky	New Siberian Islands	73.808473	142.335160	
11	Bolshoy Lykahovsky	New Siberian Islands	73.335106	141.327622	yes
12	Buor Khaya Peninsula	NE Siberia	71.689866	132.667621	
13	Bur River	NE Siberia	71.853814	122.184861	
14	Bykovsky Peninsula	NE Siberia	71.742954	129.280154	yes
15	Bykovsky Peninsula	NE Siberia	71.707809	129.014689	yes
16	Bykovsky Peninsula	NE Siberia	71.919975	129.234628	yes
17	Bykovsky Peninsula	NE Siberia	71.955863	129.146240	yes
18	Bykovsky Peninsula	NE Siberia	71.855959	129.345051	yes
19	Bykovsky Peninsula	NE Siberia	71.785093	129.411005	yes
20	Chukchi Coast	Chukotka	69.505932	177.764729	
21	Chukochya River	NE Siberia, Kolyma	69.774118	157.683984	
22	Colville River	Alaska North Slope	69.033080	-155.435873	yes
23	Colville River	Alaska North Slope	69.303220	-152.325352	yes
24	Duvanny Yar	N Siberia	68.591939	159.407355	yes
25	Duvanny Yar	NE Siberia	68.633539	159.087564	yes
26	Duvanny Yar region	NE Siberia	68.145060	159.349796	
27	East Taymyr	N Siberia	73.961032	108.722973	
28	Fadeevsky Island	New Siberian Islands	75.079517	143.796939	
29	Fadeevsky Island	New Siberian Islands	75.660481	144.565920	
30	Gydan Peninsula	Gydan Peninsula	71.007018	74.613030	
31	Gydan Peninsula	Gydan Peninsula	71.057810	74.696738	
32	Gydan Peninsula	N Siberia	72.576500	75.340480	
33	Indigirka-Kolyma lowland	NE Siberia	70.959769	156.083332	
34	Indigirka-Kolyma lowland	NE Siberia, Kolyma	69.385556	157.654533	
35	Itkillik River	Alaska North Slope	69.576221	-150.867028	yes
36	Kaolak River	North Alaska	69.987157	-159.886911	
37	Kepteni	Central Yakutia	62.803485	130.788341	
38	Khardang	NE Siberia, Lena Delta	72.950561	124.151693	
39	Khatanga River mouth	NE Siberia	73.113530	107.265371	
40	Kitepveem River	Chukotka	69.510876	164.919838	
41	Kotelny Island	New Siberian Islands	75.510802	138.839769	
42	Kotelny Island	New Siberian Islands	74.660187	139.237631	
43	Kotenly Island	New Siberian Islands	76.174295	138.989092	yes

Table S2. Cont.

44	Kukpowruk River	North Alaska	69.418498	-162.357324	
45	Kurungnakh	NE Siberia, Lena Delta	72.322869	126.252930	yes
46	Lake Portnyagino	Taymyr Peninsula	74.048820	107.333233	
47	Lake Prontshicheva	Taymyr Peninsula	75.200574	112.713298	
48	Lake Syangannakh	NE Siberia	70.678725	147.418492	
49	Lake Taymyr	Taymyr Peninsula	74.450343	101.140696	
50	Lake Ulakhan Kyuel	NE Siberia, Indigirka	70.646390	147.622238	
51	Maly Lyakhovsky	New Siberian Islands	74.070497	140.224890	yes
52	Maly Lyakhovsky	New Siberian Islands	74.167982	140.920488	
53	Mamontovy Klyk	NE Siberia	73.566473	117.356153	
54	Mamontovy Klyk	NE Siberia	73.597009	117.221389	yes
55	Mamontovy Klyk	NE Siberia	73.641158	116.749316	
56	Mys Chukochy	NE Siberia	70.066087	159.933398	
57	Mys Chukochy	NE Siberia	70.027622	159.496882	
58	Mys Chukochy	NE Siberia	70.082854	159.902104	yes
59	Mys Krestovsky	NE Siberia	70.673557	159.512780	
60	Northeast Taymyr	Taymyr Peninsula	76.377097	111.942604	
61	Northern Seward Peninsula	Alaska	66.545509	-164.454580	yes
62	Northern Seward Peninsula	Alaska	66.580383	-164.370493	yes
63	Northern Seward Peninsula	West Alaska	66.558065	-164.466410	yes
64	Novosibir Island	New Siberian Islands	75.388681	147.544096	
65	Novosibir Island	New Siberian Islands	74.772235	149.332655	
66	Oyagos Yar	NE Siberia	72.720559	141.812424	
67	Oyagos Yar	NE Siberia	72.770542	141.562778	yes
68	Point Lay	North Alaska	70.628747	-160.044900	yes
69	Selennyakh River	NE Siberia, Indigirka	68.039833	143.237817	
70	Selennyakh River	NE Siberia, Indigirka	68.032613	143.160081	
71	Shirokostan Peninsula	NE Siberia	72.295649	139.521439	
72	Sobo-Sise Island	Lena Delta	72.494451	128.051542	
73	Stadukhinsko-Plahanksi Yar	NE Siberia	68.678867	160.277539	yes
74	Stolbovoy Island	New Siberian Islands	74.086968	135.987253	yes
75	Suyalakh River	NE Siberia	71.361437	140.488497	
76	Syrdakh	Central Yakutia	62.552569	130.883758	
77	Syrdakh	Central Yakutia	62.556250	130.883795	
78	Tabaga	Central Yakutia	61.671278	130.987607	
79	Tchirovoe Lake	Chukotka	66.659236	174.338305	
80	Upper Meade River	North Alaska	70.027748	-156.947844	
81	Wrangel Island	E Siberia	70.925438	179.590992	
82	Yana-Indigirka coast	NE Siberia	72.595713	144.707206	
83	Yana Indigirka lowland	NE Siberia	71.986104	148.084983	
84	Yana River	NE Siberia	70.720286	135.382741	
85	Yukagirskiy Nasleg River	NE Siberia, Indigirka	70.321746	151.333659	

* Stadukhinsko-Plahanksi Yar is the only thermokarst-mound site in the table not identified in ESRI satellite imagery, but only through field work.

**Thermokarst mounds were found to be present by researchers G. Grosse and/or K. Walter Anthony during field expeditions since the year 1999.

Table S3. Plot-scale methane fluxes, soil moisture and soil temperature measured at chamber flux plots according to microtopographical position at 25 extensive thermokarst-mound sites on all frost-free dates (a) and at NSY on September 4-6, 2021 (n=103) and September 18-19, 2022 (n=98) (b). In rows *, different letters indicate a significant difference in methane flux among microtopographical position groups at an alpha level of 0.05 (Kruskal-Wallis rank sum test with Wilcoxon pairwise comparison).

(a) Extensive Thermokarst Mounds Microtopographical Position

	Top			Flank			Trench		
	Methane flux (mg CH_4 m^{-2} d^{-1})								
mean ± SEM	3.55	±	2.46	20.59	±	6.95	347.93	±	251.02
*	*a*			*b*			*ab*		
n	42			121			67		
median	-0.95			0.59			0		
min - max	-4.27	to	81.99	-6.49	to	574.89	-6.69	to	15,648.05
	Soil moisture at 12 cm (%)								
mean ± SEM	21.7	±	1.9	28.9	±	1.3	37.6	±	2.3
n	42			121			67		
median	20.6			31.3			37.1		
min - max	0	to	41.9	3.9	to	61.6	0.4	to	91.5
	Soil temperature at 10 cm (°C)								
mean ± SEM	8.6	±	0.3	8.5	±	0.2	8.0	±	2.4
n	42			121			67		
median	9.0			8.5			8.0		
min - max	2.0	to	12.0	1.5	to	14.0	3.0	to	13.0

(b) NSY Microtopographical Position

	Top	Flank	Trench	Water Track
	Methane flux (mg CH_4 m^{-2} d^{-1})			
mean ± SEM	7.79 ± 3.95	89.24 ± 34.14	55.64 ± 11.91	341.64 ± 235.12
*	*a*	*b*	*b*	*c*
n	31	112	45	13
median	0	4.17	7.94	81.63
min - max	-6.10 to 89.50	-13.75 to 3,206	-1.98 to 344.55	21.46 to 3,147
	Soil moisture at 12 cm (%)			
mean ± SEM	42.7 ± 0.8	44.5 ± 0.5	50.2 ± 1.6	51.1 ± 1.1
n	24.0	94.0	38.0	14.0
median	42.6	45.1	48.0	50.7
min - max	36.8 to 50.9	34.7 to 69.1	37.5 to 89.3	41.5 to 57.0
	Soil temperature at 10 cm (°C)			
mean ± SEM	9.7 ± 0.9	9.7 ± 0.2	9.7 ± 0.4	9.3 ± 0.1
n	8	25	14	5
median	9.9	10.0	10.0	9.4
min - max	6.0 to 14.4	6.0 to 10.8	5.0 to 10.7	9.1 to 9.4

Table S4a. Daily mean temperature, moisture, and methane and carbon dioxide fluxes at the NSY eddy covariance tower for specific seasons. * indicates a partial season record. Mean daily methane emissions summarized by type of season were 178.4 ± 8.0 mg CH_4 m^{-2} d^{-1} (mean ± SEM), n=77 thaw season days; 55.7 ± 2.3 mg CH_4 m^{-2} d^{-1}, n=274 summer days; 165.8 ± 4.0 mg CH_4 m^{-2} d^{-1}, n=387 winter days. Methane and carbon dioxide fluxes differed by season within study years, Kruskal-Wallis rank sum: CH_4: $X^2(6)=401.8$, p<.001; CO_2: $X^2(6)=161.4$, p<.001; *different letters indicate significant differences at an alpha level of 0.05 based on the Wilcoxon rank sum pairwise comparison test with continuity correction.

Season	THAW*	SUMMER	WINTER	THAW	SUMMER	WINTER	THAW*
Date	5/8/21 - 6/7/21	6/8/21 - 10/17/21	10/18/21 - 5/2/22	5/3/22 - 6/8/22	6/9/22 - 10/28/22	10/29/22 - 5/6/23	5/7/23 - 5/15/23
Air temperature (°C)							
mean ± SEM	11.0 ± 0.5	10.1 ± 0.6	-12.7 ± 0.8	10.2 ± 0.9	9.8 ± 0.6	-13.0 ± 0.6	8.5 ± 0.7
n	31	132	197	37	142	190	9
(min-max)	4.3 to 15.1	-6.5 to 22.1	-37.2 to 7.5	1.2 to 19.4	-16.6 to 20.4	-36.2 to 7.9	5.4 to 11.9
Surface soil temperature (°C)							
mean ± SEM	ND	7.4 ± 0.5	-1.2 ± 0.1	4.7 ± 0.5	9.4 ± 0.4	-0.5 ± 0.0	1.0 ± 0.2
n		74	197	37	142	184	9
(min-max)		0.2 to 16.9	-4.1 to 0.0	0.2 to 9.4	0.0 to 15.7	-1.5 to 0.0	0.1 to 2.2
Soil temperature at 10 cm (°C)							
mean ± SEM	4.0 ± 0.4	9.8 ± 0.4	-0.9 ± 0.0	2.5 ± 0.4	9.0 ± 0.3	-0.2 ± 0.0	-0.1 ± 0.0
n	19	132	197	37	142	184	9
(min-max)	2.1 to 6.5	1.0 to 15.1	-2.4 to 0.7	-0.3 to 6.9	1.0 to 14.0	-0.5 to 0.8	-0.2 to 0.0
Soil moisture (%)							
mean ± SEM	32% ± 0%	32% ± 0%	31% ± 5%	29% ± 1%	29% ± 0%	28% ± 0%	24% ± 0%
n	19	132	30	25	142	24	2
(min-max)	28% to 33%	29% to 34%	20% to 32%	22% to 31%	28% to 31%	28% to 29%	24% to 24%
Methane flux (mg CH_4 m^{-2} d^{-1})							
mean ± SEM	187.5 ± 15.6	77.5 ± 2.7	152.2 ± 4.4	158.8 ± 11.0	35.4 ± 2.6	180.0 ± 6.7	227.6 ± 26.1
*	*a*	*b*	*c,e*	*a,c*	*d*	*a,e*	*e*
n	31	132	197	37	142	190	9
median	182.9	78.4	139.2	145.4	26.3	162.0	261.9
(min-max)	49.1 to 380.6	17.8 to 162.8	31.9 to 351.3	47.6 to 338.4	5.5 to 151.5	42.8 to 423.0	114.8 to 321.3
Carbon dioxide flux (g CO_2 m^{-2} d^{-1})							
mean ± SEM	6.7 ± 0.5	3.1 ± 0.4	2.8 ± 0.1	3.2 ± 0.8	-0.6 ± 0.6	6.0 ± 0.2	6.5 ± 0.7
*	*a*	*b*	*b*	*b*	*c*	*a*	*a*
n	31	132	197	37	142	190	9
median	6.8	3.4	2.7	3.1	-2.1	5.4	6.0
(min-max)	2.5 to 12.8	-11.4 to 13.1	-3.4 to 11.0	-6.9 to 15.8	-17.0 to 19.3	0.6 to 16.8	3.6 to 10.8

Table S4b. Summary of phenologically-determined seasonal and annual methane and carbon dioxide fluxes at the NSY eddy covariance tower. Methane and carbon dioxide fluxes were higher in Year 1 compared to Year 2, Kruskal-Wallis rank sum: CH_4: $X^2(1)=10.3$, p=.001; CO_2: $X^2(1)=4.9$, p=.03. Methane flux expressed on a carbon-mass basis in CO_2-equivalents was calculated based on GWP_{100} of 28.

	Year 1<br>5/10/21-5/13/22	Year 2<br>5/14/22-5/14/23
	Methane flux (g CH_4 m^{-2} yr^{-1})	
Total annual	47.9	44.8
Summer	22%	10%
Winter	62%	76%
Thaw	16%	14%
	Carbon dioxide flux (g CO_2 m^{-2} yr^{-1})	
Total annual	1254.3	1141.1
Summer	33%	-2%
Winter	44%	100%
Thaw	23%	2%
	Methane flux (g C-CO2e m^{-2} yr^{-1})	
	366.1	*342.3*
	Carbon dioxide flux (g C-CO_2 m^{-2} yr^{-1})	
	342.1	311.2
	Methane contribution to warming	
	52%	52%

Table S5. Overview of key model parameters and values. For the model sensitivty tests, one parameter has been varied at a time, while the others remained at their default values. Note that the tile areal fractions and tile initial elevations have been varied in combination.

**Parameter(s)**	**Symbol**	**Unit**	**Value (default bold)**
Tile areal fractions	$\gamma_{M \mid F \mid T}$	-	**0.3 | 0.6 | 0.1**, 0.3 | 0.4 | 0.3
Reservoir hydraulic conductivity	K_{res}	$m\ s^{-1}$	$\mathbf{2\pi\ 10^{-5}}$, $2\pi\ 10^{-6}$
Reservoir elevation	e_{res}	m	**-10.0**
Tile initial elevation	$e_{F \mid T}$	m	**0.0 | 0.0**, -1.0 | -2.0
Snow multiplier and snow density	φ	-	**1.0**, 1.5
Snow density	ρ	$kg\ m^{-3}$	**250**, 200

Table S6. Overview of the (initial) ground stratigraphies. Layers containing excess ice are highlighted in bold font.

**Depth [m]**	**Mineral content**	**Organic content**	**Natural porosity**	**Soil type**	**Water/ice content**	**Comment**
Mound tops						
0-0.1	0.00	0.15	0.85	sand	0.85	Vegetation layer
0.1-0.2	0.10	0.15	0.75	sand	0.75	Organic layer
0.2-0.6	0.25	0.10	0.65	silt	0.65	Mineral layer
**0.6-20.0**	**0.25**	**0.10**	**0.55**	**sand**	**0.65**	Ice-rich deposits
20.0-30.0	0.35	0.10	0.55	sand	0.55	Younger taberite deposits
30.0-50.0	0.50	0.05	0.45	sand	0.45	Older taberite deposits
>50.0	0.90	0.00	0.10	sand	0.10	Bedrock
Mound flanks						
0-0.1	0.00	0.15	0.85	sand	0.85	Vegetation layer
0.1-0.2	0.10	0.15	0.75	sand	0.75	Organic layer
0.2-0.6	0.25	0.10	0.65	silt	0.65	Mineral layer
**0.6-1.0**	**0.25**	**0.10**	**0.55**	**sand**	**0.65**	Intermediate layer
**1.0-20.0**	**0.10**	**0.05**	**0.55**	**sand**	**0.85**	Ice wedge and ice-rich deposits
20.0-30.0	0.35	0.10	0.55	sand	0.55	Younger taberite deposits
30.0-50.0	0.50	0.05	0.45	sand	0.45	Older taberite deposits
>50.0	0.90	0.00	0.10	sand	0.10	Bedrock
Trenches						
0-0.1	0.00	0.15	0.85	sand	0.85	Vegetation layer
0.1-0.2	0.10	0.15	0.75	sand	0.75	Organic layer
0.2-0.6	0.25	0.10	0.65	silt	0.65	Mineral layer
**0.6-1.0**	**0.25**	**0.10**	**0.55**	**sand**	**0.65**	Intermediate layer
**1.0-20.0**	**0.05**	**0.00**	**0.55**	**sand**	**0.95**	Ice wedge
20.0-30.0	0.35	0.10	0.55	sand	0.55	Younger taberite deposits
30.0-50.0	0.50	0.05	0.45	sand	0.45	Older taberite deposits
>50.0	0.90	0.00	0.10	sand	0.10	Bedrock

3. Supplementary Methods

3.1 *Geophysics*

Electrical resistivity tomography (ERT) measurements are a useful tool for characterizing permafrost distributions, particularly when they are combined with downhole nuclear magnetic resonance (NMR) measurements that estimate in situ volumetric water content (VWC) with depth[4-6]. Electrical resistivity is very sensitive to the presence of even a small amount of liquid water, such as in thawed talik zones or even within permafrost that has warmed or contains solutes[7,8]. Soil resistivity values are highly dependent on temperature and soil moisture. Dry and frozen soils exhibit much higher electrical resistivity values compared to wet, thawed soils. Sediment grain size (e.g., silt vs. gravel) can also influence electrical resistivity, with smaller grainsizes typically exhibiting lower electrical resistivity[6,9].

Ground-based ERT data were collected at NSY along two near-perpendicular transects (Fig. S2) using an AGI SuperSting R8 system (Advanced Geosciences, Inc., Austin, Texas) on September 15, 2021[3]. Survey lines comprised 112 stainless steel electrodes at 2 m spacing for a total length of 222 m. The North Star A transect ran from northeast to southwest across the cleared field, starting next to a small pond at the edge of the field and ending in the forest where a large trench was crossed. The North Star D profile ran downslope, starting in the forest at the south edge of the field and ending just north of the intersection with the North Star A transect. The locations and orientations of the ERT transects were chosen to maximize overlap with chamber measurement locations.

Electrical resistivity data were acquired using a dipole-dipole array, and data were inverted using ResIPy inversion software (version 3.3.2)[10] to produce 2-D profiles of resistivity along each transect (Fig. S2c,d). Results are shaded with a white overlay at depth in areas of low data sensitivity where model parameter uncertainty is greater.

Borehole NMR data were collected in BH1 between 0.25 – 2.25 m depths, with 0.25 m vertical sampling intervals using a Dart in situ soil moisture probe (Vista Clara, Inc., Mukilteo, Washington). NMR data were analyzed using proprietary software [Vista Clara acquisition (7.1.8) and processing (3.62/1.64) software (Vista Clara, Inc, Mukilteo, WA, USA]) to produce estimates of total water content and pore size distribution based on multiexponential fitting to the measured T2 decay curve data[11]. Total volumetric liquid water content for the mobile, capillary, and bound components of the pore space was derived from the integrated partial water contents (water content as a function of T2 decay time constant, computed from the multiexponential fits) with time constant values less than 3.16 ms, between 3.16 ms and 31.6 ms, and greater than 31.6 ms, respectively (Fig. S2b).

Lastly, electrical resistivity values were transformed to estimates of water content (Fig. 4) using an empirical relationship derived from the co-located ERT and NMR measurements following the approach described in Minsley et al.[12]. Parameters for the resistivity-water content relationship were adjusted slightly to accommodate the single ERT-NMR calibration point at NSY.

3.2 *Borehole sediment sampling and analyses*

We sampled NSY soils in summer and winter by drilling boreholes, BH1 and BH6, on September 15, 2021 and March 18, 2023, respectively. These boreholes, 60-cm apart, were located on top of a thermokarst mound at mid elevation in the NSY study field adjacent to the eddy covariance tower. BH1 was drilled with a gas-powered AMS frozen soil auger with 5 cm of internal (core) diameter (https://www.ams-samplers.com/frozen-soil-auger-kit/). Except for the

porewater gas analyses, soil subsampling at 10 to 15-cm intervals along the 3.2-m BH1 core was not volumetrically controlled. Soil samples were placed in Ziploc bags, and transferred to the freezer. In winter we collected the BH6 core using a Talon Drill system, which enabled us quantify soil dry density in 56 samples in volumetrically-controlled samples distributed throughout the ~7.0 m deep talik and underlying permafrost soil down to 7.25 m. Here talik thickness was similar to that (6.8 m) determined by drilling on June 6, 2022 several meters away. On June 6, 2022 four soil pits were dug at different microtopographical locations (mount top, flanks, hollow) near BH1 to determine depth of seasonal frost (≤ 85 cm). Soil samples were collected and subsamples exported in a responsible manner and in accordance with relevant permits and local laws.

In the lab, we determined gravimetric soil moisture content as weight loss dried at 105 °C expressed as percentage of wet weight. We calculated volumetric moisture content of talik soils as the product of gravimetric moisture content and dry density. Since dry density was not measured on BH1, we used a depth-dependent dry density relationship determined from BH6. Percent water saturation, which is the ratio of the total volume of voids to the volume of free water in a sample, was calculated as the product of water content and specific gravity divided by the void ratio. We adjusted the specific gravity of silt with low organics (2.65) to account for organic matter concentration, calculated as 1.8 times the measured organic carbon concentration[13].

Wood fragments extracted from three depths in the BH1 core were radiocarbon dated using ^{14}C Accelerator Mass Spectrometer (AMS) at the Woods Hole Oceanographic Institution's National Ocean Sciences AMS (NOSAMS) Facility[14].

3.3 *Soil dissolved gas concentrations and isotopes*

To determine dissolved CH_4 and CO_2 concentrations and the stable carbon isotopic composition ($\delta^{13}C$) of CH_4 and CO_2, a 3 ml soil plug was collected with a tip-cut 5 ml syringe from each depth section of the BH1 and BH6 cores and placed in a 20 ml glass serum containing preboiled and cooled 5M NaCl solution (inhibiting microbial activity). The vials were closed immediately with butyl rubber stoppers (GMT) and aluminum crimp caps with no headspace (this was achieved by inserting a needle into the stopper, while it was closed, allowing some of the salt water out, making some room for the relatively massive stopper), and then stored upside down until processing.

In the lab, a 3 mL gas headspace at atmospheric pressure was created in each porewater sample vial by addition of ultrapure nitrogen using two sterile syringes. Samples were vigorously shaken for two minutes and analyzed for CH_4 concentration. After two weeks equilibration at 22 ºC, we measured the CO_2 and O_2 concentration in samples. Porewater dissolved CH_4, CO_2 and O_2 concentrations were calculated according to the ideal gas law, considering Henry's constants (K_H) in the salt solution using data from Stumm and Morgan[15], USGS PHREEQC v.3[16], and Bok et al.[17].

Methane concentration was analyzed on a Focus Gas Chromatograph (GC) system (Thermoscientific, Germany) equipped with a flame ionization detector and shinCarbon ST packed column (Restek, USA). Injection volume was 240 µL. O_2 concentration was measured by injecting 1 mL of diluted headspace into a 10 mL serum vial crimped with blue silicon septa, flushed with ultrapure helium. The vial was fitted with a PSt6 sensor spot that was monitored by Fibox3 (Presens, Germany), capable of measuring O_2 at ppb levels (1% O_2 mix was used for calibration of the sensor spot).

The $\delta^{13}C$ of CH_4 and CO_2 values and CO_2 concentrations were determined using Gas Source Isotopic Ratio Mass Spectrometer (GS-IRMS) interfaced with a preconcentrating device (PreCon) and Gas Bench II (Thermo Scientific, Germany), equipped with 1 mL sample loop and Pora plot GC (Rt-Q-Bond, fused silica PLOT Restek, USA) for CO_2 separation. Samples of 100 to 1000 µL were injected into ultrapure helium flushed vials (12 mL, Labco UK) to be measured. All gas standards had been calibrated against reference materials from NIST 8559, 8560, and 8561 (for CH_4) or NBS-19 and NBS-18 (for CO_2), using the GS-IRMS, or were provided from Air Gas (Air Liquide, Plumsteadville, PA, USA) or from Isometric Instruments (GASCo, Victoria, BC, Canada) with certificates. Isotope values are expressed in units of per mill (‰) in the typical δ-notation vs. V-PDB.

3.4 *DNA extraction, 16S rRNA gene V4 amplicon-sequencing*

Sediment samples from the summer core (BH1: 23 cm, 56 cm, 68 cm, 86 cm, 106 cm, 166 cm, 186 cm, 259 cm, 295 cm and 305 cm) and winter (BH6: 50 cm, 100 cm, 162 cm, 198 cm, 285 cm, 345 cm, 535 cm, 564 cm, 655 cm, 695 cm and 711 cm), were chosen for amplicon-based sequencing. DNA extraction (approximately 0.25 gr) was performed using the DNeasy PowerSoil Pro Kit (QIAGEN, Hilden, Germany; prior MoBio, CA, USA; Cat. No. 47016), according to the manufacturer's instructions. DNA extracts were subsequently stored at -80 °C.

The gene expression of *mcrA* and *pmoA*, was assessed from the DNA extractions by qPCR, utilizing primer sets ME1F (forward, 5'-GCMATGCARATHGGWATGTC-3') and ME3R (reverse, 5'-TGTGTGAASCCKACDCCACC-3') for the *mcrA* gene[18,19] and A189gc (forward, 5'-GGNGACTGGGACTTCTGG-3') and mb661 (reverse, 5'-CCGGMGCAACGTCYTTACC-3') for the *pmoA* gene[20-22]. Primers were purchased from Sigma-Aldridge, Israel. qPCR reactions were run in triplicates, each, in a total volume of 20 µl, containing 10 µl Fast SYBR™ Green Master Mix (Applied Biosystems™, ; Cat. No. 4385612). Forward and reverse primer concentrations were 0.25 mM for the *mcrA* gene and 0.5 mM for the *pmoA*. Reactions were performed using Rotor Gene 6000, software version 1.7 (Corbett Research, UK), with the following conditions: 20 sec activation at 95 °C, followed by 40 cycles of denaturation of 95 °C for 3 sec, annealing of 53 °C (*mcrA*) and 56 °C (*pmoA*) for 30 sec and an extension step of 30 sec at 72 °C. gBlocks dsDNA fragments were used as standard calibration curves for *mcrA* (*Methanolacinia petrolearia* DSM 11571; GenBank accession number: NC_014507.1) and *pmoA* (*Methylococcus capsulatus* particulate methane monooxygenase 27 kDa subunit (*pmoA*); GenBank accession number: L40804.2) (Integrated DNA Technologies, Coralville, IA, USA). Standard calibration curves with known concentration of the target gene (gBlocks) vs cycle threshold (CT) were used to determine gene concentration.

16S gene amplicon-based sequencing (targeting the V4 region), was performed using the modified primer pair with consensus sequences CS1_515F (ACACTGACGACATGGTTCTACAGTGCCAGCMGCCGCGGTAA) and CS2_806R (TACGGTAGCAGAGACTTGGTCTGGACTACHVGGGTWTCTAAT) (Sigma-Aldridge, Israel)[23]. The first PCR was performed in 25 µl reactions, containing 12.5 µl KAPA HiFi HotStart ReadyMix (KAPA Biosystems, Wilmington, WA, USA; Cat. No. 07958935001), 0.75 µl forward and reverse primers at a final concentration of 300 nM each. PCR conditions were: an initial denaturation at 95 °C for 3 min, followed by 30 cycles of 98 °C for 20 sec, 60 °C for 15 sec and 72 °C for 30 sec. PCR products were inspected on a 2% agarose gel to measure bands relative intensity. Samples were pooled and purified using calibrated Ampure XP beads and used for library preparation. PCR visualization, purification, library preparation and sequencing (2 ×

250 bp pair-end reads) were performed at HyLabs (Israel), and sequenced on an Illumina MiSeq platform. Demultiplexing of the paired end reads and subsequent analysis was done using QIIME2 V.2020-11[24]. Quality of sequenced reads was assessed using the q2-demux plugin, followed by chimera detection, and merging of reads into Amplicon Sequence Variants (ASVs) with DADA2[25], using the q2-dada2 plugin. To account for length variations, ASVs were defined by clustering at 100% similarity[26]. Taxonomy was assigned using the QIIME-release of the 138-SILVA database (99% clustering)[27]. Extract-reads and fit-classifier-naive-bayes methods were used for the classifier, via the q2-feature-classifier plugin[28] and classification of the ASVs by the classify-sklearn method (version 0.23.1)[29]. Downstream analyses were performed in R (version 4.3.2), using the packages phyloseq[30], and ggplot2[31].

3.5 *Remote-sensing detection of thermokarst mounds in the pan-Arctic*

The very high-resolution ESRI satellite basemap, consisting of recent imagery from SPOT and MAXAR satellites for the period 2020-2022, was used to identify locations with thermokarst mounds in the pan-arctic region. First, we selected sites known to have thermokarst mounds based on our own field experience from expeditions between 1999-2023 (Grosse, Walter Anthony) and confirmed the usually very good visibility of the mounds in the very high-resolution imagery (0.5 m) for tundra areas. An additional random stratified sampling for thermokarst-mound presence was conducted across the Arctic in regions where large ice wedge polygons were visible. If thermokarst mounds in larger numbers were encountered in a region, a point was placed in a point shapefile at a typical mound site. Overall, thermokarst mounds occurred in different settings including extensive flat uplands, hillslope areas, gullies and valley networks, river banks, as well as retrogressive thaw slumps on coasts, rivers, and lakes. The 85 mapped sites are exemplary and not exhaustive.

3.6 *Numerical Modeling*

We used the CryoGrid3 permafrost model[32-34] to simulate the geomorphological and hydrothermal evolution of well-drained Yedoma uplands in northeastern Siberia through the 21st and 22nd centuries. We followed Nitzbon et al.[34] to set up a model configuration in which three model tiles represent the surface and subsurface heterogeneities corresponding to thermokarst mound tops, flanks, and trenches (Fig. S10). For each model tile, the model represents the vertical heat transport with phase change of water, water infiltration, and ground subsidence due to melting of excess ground ice. The tiles are laterally coupled through transport of heat, water, snow, and sediment, and water can drain from the model domain via the trenches. We used the same meteorological forcing data as Nitzbon et al.[34] for the central Lena river delta according to the strong climate warming scenario RCP8.5. The forcing timeseries were extended through the 22nd century to represent climate stabilization by looping five times through the 2080-2100 period. The ground stratigraphies and further model parameters were set up to reproduce the well-drained Yedoma deposits setting by Nitzbon et al.[34] (Table S6). In addition, we tested the sensitivity of the model within plausible yet uncertain parameter ranges related to snow conditions, thermokarst-mound geometry, initial topography, and drainage efficiency (Table S5). From the simulated annual minimum/maximum ground temperatures ($T^{\mathrm{min/max}}$) and annual minimum liquid water contents (θ^{min}), we diagnosed the hydrothermal state as one of the following:permafrost ($T^{\mathrm{max}}<0°C$, $\theta^{\mathrm{min}}<0.10$),

(i) freeze-thaw ($T^{max}\geq 0°C$, $\theta^{\mathrm{min}}<0.10$),

(ii) cryotic talik (T^{min}<0°C, θ^{min}≥0.10), or
(iii) non-cryotic talik (T^{min}>0°C, θ^{min}≥0.10).

4. Supplementary Results

4.1 *Soil management effects*

Chamber flux measurements conducted at heavily-managed and unmanaged thermokarst mounds at the Isabella study site on September 19, 2023 showed no effect of soil management on methane fluxes. Thermokarst mounds formed following spruce-forest clearing in the 1940s. During our 2021-2022 study period, mounds were covered by grasses and vetch with some shrubs. During the summer of 2023, the landowner heavily disturbed half of the thermokarst-mound study field. Disturbance included mowing, bushwhacking, grinding roots and stumps, bulldozing to level the mounds, discing and rototilling ≤10 cm of the ground surface, dragging to smooth the ground surface, compaction, fertilization and seeding with lawn grasses. We conducted chamber flux measurements approximately four weeks after following the disturbance. A Kruskal-Wallis rank sum test indicated no significant difference in methane flux between unmanaged thermokarst-mounds and areas heavily impacted by soil and vegetation management, $X^2(1)=0.171$, p=.679.

4.2 *Surface soil moisture and methane*

The wider range of soil moisture conditions among the extensive study sites is explained by their highly variable geographic (ecological, latitudinal and climatic) characteristics (Table S1), while within a single site (i.e. NSY) moisture is more tightly constrained. Despite differences in surface soil moisture, the occurrence of net positive methane emissions from thermokarst mound sites underscores the importance of deep taliks as environments for methane production and transport. The observation of high fluxes even at very low VWC is evidence that dry soils allow transmission of gas produced deeper within the talik to the atmosphere.

Moisture thresholds for microbial methane consumption also surfaced in our analysis. Among 316 paired observations of surface soil moisture coincident with chamber-based methane fluxes at NSY, substantially positive methane fluxes (>50 mg CH_4 m^{-2} d^{-1}) were not observed at VWC<37%, while all zero or negative methane fluxes were observed at VWC<53%. Among 275 sets of observations at our extensive thermokarst-mound study sites, substantially positive methane fluxes (>50 mg CH_4 m^{-2} d^{-1}) were observed at VWC as low as <5%; however, all negative and 61 out of 64 zero methane fluxes were observed at VWC<50%. These results indicate that surface soil moisture impacts methanotrophy and ~50% VWC appeared to be a threshold above which net negative fluxes were not observed.

5. Supplementary Discussion

5.1 *Impedances to talik methane emissions*

High methane (CH_4) fluxes from subaerial taliks to the atmosphere were impeded by several factors including: (1) A mid-winter rain on snow event on December 26, 2021, (2) seasonal frost, and (3) aerobic methane oxidation in summer.

First, a December 27, 2021 rain on snow event created a ~4 cm layer of ice in the snowpack. Careful removal of this ice layer with a snow shovel in February and March 2022 at thirteen ~720 cm^2 NSY chamber plots resulted in 2 to 70-times higher fluxes (mean=15) in chambers (Wilcoxon signed rank test, p=.002), indicating that methane emitted from the soil

surface was being trapped by the ice-in-snow layer. This snow-ice layer was not a perfect seal; however, since emissions were still positive from the undisturbed snow prior to ice removal and high emissions measured by the EC tower and chambers continued through the winter snow cover period.

A second impedance to deep-sourced terrestrial methane emissions was seasonal frost. On June 6, 2022, a time when snow had completely melted, field grasses had begun to grow, and surface soils had thawed down to 30 to 50 cm, we drilled through the remnant layer of surface seasonal frost (~10 to 30 cm thick) sandwiched between the surface thaw and underlying talik at three locations on a thermokarst mound (top, flank, trench) near the NSY tower. Chamber flux measurements over the boreholes penetrating seasonal frost at the top and flank showed 11,000 to 33,000-times higher fluxes (up to 94,343 ± 4,252 mg CH_4 m^{-2} d^{-1}, mean ± SEM, n=2) compared to the undisturbed ground surface prior to drilling (3 mg CH_4 m^{-2} d^{-1}, n=1(Fig. 6c). In the trench, we observed the shallowest thaw depth (32 cm), little (<10 cm) seasonal frost, and emissions from the undisturbed surface an order of magnitude higher (35 ± 17 mg CH_4 m^{-2} d^{-1}, n=3) than the top and flank sites. When we penetrated seasonal frost in the trench with a drill, borehole emissions shot up to 8,559,845 ± 739,362 mg CH_4 m^{-2} d^{-1}, which was 245,600 times higher than from the undisturbed trench surface prior to drilling. A Kruskal-Wallis rank sum test indicated significantly higher methane flux from depths below the seasonal frost layer ($p<.001$). Analysis of variance of a linear model of log-transformed methane flux showed a significant relationship between flux and borehole depth ($p<.001$), with no significant microtopographical effect or borehole-depth interaction. We attribute the shallow, thin frost layer, through which talik methane was migrating in the trench to microtopographical controls on snow accumulation in winter. Redistribution of snow from thermokarst-mound tops to trenches leads to an insulating effect in trenches, protecting against deep seasonal frost in winter and leading to slower melt and ground thaw in spring. Conversely, less snow on thermokarst-mound tops leads to deeper seasonal frost, but more rapid snowmelt and ground surface thaw in spring. Our observations of snow thickness at NSY in March 2023 support this hypothesis. Snow was 5 to 18 cm deeper in trenches than mound tops and shoulders. Since dense boreal forest surrounding NSY protect it from strong winds, we expect snow redistribution across thermokarst-mound microtopography to be even stronger in windy tundra, a phenomenon we have experienced in field work (G. Grosse pers. comm., Nov. 21, 2023).

The six- to seven-order-of-magnitude increases in methane emissions following penetration of the seasonal frost layer at all thermokarst-mound positions on June 6, 2022 (Fig. 6c), and observations of the highest plot-scale emissions (≤12,790 mg CH_4 m^{-2} d^{-1}) occurring during spring thaw from among 663 year-round measurements of undisturbed NSY ground surface fluxes (Table S1b) suggests seasonal frost acts as a partial seal to methane produced in the underlying talik and that this methane is released in spring during thaw of the season frost layer. However, this impeding seasonal frost layer was not a totally impenetrable barrier to deep-sourced methane. We observed high wintertime emissions (≤1,427 mg CH_4 m^{-2} d^{-1}) from several undisturbed locations in our chambers (Table S1b), and tower fluxes (168 ± 4 mg CH_4 m^{-2} d^{-1}, mean ± SEM, n=464; 32 to 423 mg CH_4 m^{-2} d^{-1}, min to max), which integrated the myriad of fluxes observed across the field surface throughout the winter and thaw periods, were positive.

Third, arguably the most important impedance to methane emissions from taliks on an annual basis was microbial aerobic oxidation in dry surface soils. NSY eddy covariance tower emissions were more than two times lower in summer 2022 compared to 2021. Lower precipitation in summer 2022 (104 mm) compared to summer 2021 (162 mm), despite similar

summer temperatures (Table S4a), would lead to drier soil conditions that enhance aerobic methanotrophy[35,36]. While removing surface soils in summer 2022 through drilling boreholes and digging soil pits, we discovered that in most locations soil methane concentrations were still exceedingly high in the mineral soils beneath the surface at thermokarst mound sites, but not at control sites (Figs. 6a,c). Two-way ANOVA revealed a significant interaction between site type (thermokarst mounds vs. controls) and depth on borehole methane flux ($F(1,28)=5.58$, $p=.03$). Among thermokarst-mound boreholes, the site-depth interaction had a significant impact on methane fluxes (Two-way ANOVA, $F(11,8)=3.62$, $p=.04$). Methane emissions emission increase with depth in thermokarst-mound boreholes ($p=.001$). In contrast, fluxes in the control-site boreholes were negative and trended more negative with depth. For soil pits, we found a significant interaction between site type (thermokarst mounds vs. controls) and depth on methane flux ($F(1,69)=4.60$, $p=.04$). Among thermokarst mounds, the site-depth interaction had a significant impact on soil-pit methane fluxes (Two-way ANOVA, $F(17,24)=3.26$, $p=.004$). Methane emission varied by soil-pit site and increased with depth within sites ($p<.001$). Collapse of soil pores and possible creation of new fractures in the matrix by our disturbances may have contributed to some of the elevated fluxes, but the disturbance itself did not introduce any directional bias in fluxes. This is evident from seven control site observations where we did not observe an increase in methane flux with depth following disturbance to surface soils. Also, among the thermokarst-mound soil pits, occasionally we observed a decrease (not increase) in flux. Altogether, the observations imply that soil methane concentrations increase with depth in thermokarst-mound taliks and that surface soils act as a strong biofilter. Despite this powerful capacity of methanotrophy to limit net emissions, belowground methane still found escape routes through preferential flow pathways in summer (Fig. 7a), and especially during winter (Fig. 7b), resulting in net positive emissions from upland thermokarst-mound environments year-round.

These findings have global implications. Microbial methane oxidation is a strong[36], but not perfect filter. Oh et al.[36] showed that accounting for microbial methane oxidation helps reconcile the difference between process-based models which over-estimate arctic methane emission by 5-10 Tg CH_4 yr^{-1} when compared with observation-based atmospheric inversions. Oh et al.[36] also showed that negative feedbacks between temperature and HAMS suppress the positive feedback predicted to occur between temperature and methane emission due to more accessible soil organic carbon from thawing permafrost. Our findings agree that microbial methane oxidation is important in summer; however, methane still escapes from preferential flow paths that bypass oxidation in summer and to an even greater degree in winter. This implies that simulations of 21st century methanotrophic compensation of enhanced permafrost-carbon derived methanogenesis may be overestimated. Furthermore, the main energy source for HAM (methane) is assumed to be the atmosphere, and is thought to reduce in concentration with soil depth as a result of diffusivity. Our work shows that the methane energy source for surface soil methanotrophy comes not only from the atmosphere, but also from underlying upland talik soils and that it increases (not decreases) with depth.

5.2 *Enhancements of talik methane emissions*

Processes which we found to enhance methane emissions at NSY were rainfall and barometric pressure pumping. In both summers 2021 and 2022, methane emissions increased following summer rain events due to surface soil moisture displacing oxygen from soil pores and slowing oxygen diffusion into soils (Fig. S6). Fluctuation in barometric pressure also influenced methane flux at NSY, as it has been shown to do in a variety of other systems including lakes

and peatlands[37-39], landfills[40,41], unsaturated vadose zones of natural gas fields[42]. Due to pressure gradients between the atmosphere and subsurface, upward gas migration is limited during periods of high barometric pressure. Conversely, periods of low barometric pressure enhance advective transport of belowground gas to the atmosphere through preferential flow pathways in desaturated pore channels[43]. At NSY's subaerial talik site, we found an inverse relationship between changes in atmospheric pressure and methane flux among a selection of 30 prominent episodes of decreasing pressure (Fig. S8). Pressure-driven emissions were highest in winter, but also contributed to methane transport bypassing methanotrophy in surface soils in summer (Fig. S8). To avoid any potential biases associated with our selection of pressure change episodes, we also explored the relationship between pressure and methane across a continuous set of observations. We marked inflection points of barometric pressure changes from January 11, 2022 through May 3, 2022 and found a 1:1 relationship with the timing of methane flux increase or decrease (Fig. S9b). This strong correlation demonstrates the direct influence of barometric pressure on thermokarst-mound methane emissions. Furthermore, we segregated the continuous dataset into intervals defined as increasing or decreasing barometric pressure trends (Fig. S9a) and plotted the cumulative deviation in pressure and methane flux within the discrete intervals representing fully time-integrated changes (Fig. S9c). Two-way ANOVA revealed no significant interaction between the direction of barometric pressure change (positive vs negative) and cumulative pressure deviation on the cumulative deviation in methane flux ($F(1,14)=0.32$, $p=.61$). A subsequent linear regression model indicated a positive relationship between cumulative deviations in pressure and flux ($p<.0001$). While this significant relationship indicates that magnitude and duration of the pressure cycle influences methane emissions, the correlation was not as strong as for timing alone (Fig. 9b). Some potential factors that can affect magnitude of emissions response to barometric pressure change include in situ sediment mechanics[43] and hysteresis from depletion of gas storage inventories belowground from a recent pressure drop[42]. Future work could benefit from wavelet analysis to incorporate variations in fluxes due factors that act on different time scales such as barometric pressure, temperature, precipitation, and seasons.

Based on these observations of impediments and enhancers of subaerial talik methane emissions, we anticipate several biophysically-driven negative feedbacks to climate in upland thermokarst methane systems. Deeper, longer seasonal freeze has the capacity to reduce aerobic methanotrophy in surface soils and enhance anaerobic conditions in taliks. This will lead to higher net annual emissions compared to years with shorter, warmer winters. Conversely, dry summers have the potential to increase methanotrophy in surface soils. More rigorous work could help to understand the biophysical and biogeochemical controls on methanotrophy in upland soils and time scales over which factors such as precipitation, temperature, thermal convection, and barometric pressure control emissions. Soil respirometric assays would also be useful to determine temperature, redox and nutrient controls on methanogenesis and methanotrophy in upland taliks.

5.3 *Geophysical inference of permafrost, water content, and relationship to gas flux observations*

A comparison of September 2021 chamber-based methane fluxes to geophysically-determined VWC revealed that methane flux was primarily driven by maximum water content at depth in soils; however, soil-column moisture variability (i.e., ratio of shallow to deep VWC) gave rise to differences in fluxes that are most likely explained by heterogeneity in gas transport pathways and aerobic methane oxidation. At the field scale, locations of high methane flux and

high VWC of talik soils occurred with greater frequency lower on the hillslope (Fig. 4b). Here we describe the details of these findings.

Electrical Resistivity Tomography (ERT) results showed consistently low resistivity (12 – 30 ohm-m) in the uppermost several meters, consistent with fully thawed soils and the measured permafrost depth of 6.8 m to 7 m at the site (Fig. S2). Resistivity values began to increase sharply starting around 5 m depth, rising to around 150 – 1000 ohm-m beneath 10 m. This increase in resistivity was attributed to decreasing liquid water content at the permafrost table, also consistent with soil core measurements from BH6 (Fig. 5b). Given the observed resistivity of approximately 50 ohm-m at 6.8 m depth nearest BH6 where permafrost was identified (Fig. S2e, yellow star), we infer depth to permafrost around 5 – 9 m at other locations along the ERT transects (Fig. S2e, red line). Using shallow soil VWC and NMR water content estimates co-located with ERT transects, we developed a site-specific relationship to transform resistivity values to VWC along our profiles (Fig. S3) following the approached described in Minsley et al.[12] with parameters adjusted to fit limited borehole observations at this study site.

We assessed relations between plot-scale observations of methane fluxes and microtopography with estimates of resistivity and geophysically-derived soil VWC using regression models. Our model database was constructed using methane flux observations that were collected in September 2021 and those within 2 m of our ERT transects (n=36). Geophysical metrics representing soil conditions (i.e., resistivity, VWC) and vertical gradients (e.g., mean, max, ratios) were systematically correlated with methane fluxes on both log and linear scales. Our objective was to develop an accurate and interpretable model with constraints imposed given the limited sample size. Due to the low number of zero and negative fluxes recorded within 2 m of the ERT transects—n=4 and n=2, respectively—relationships were dominantly driven by the positive methane fluxes. The log-transformed positive methane fluxes exhibited the best model fit to the maximum VWC in the top 3 meters, scaled by the ratio between the mean VWC in the upper 3 m relative to the mean VWC in the upper 1 m (labeled vertically weighted maximum water content) (r==.64; r^2=.41; p-value <.0001; slope: 3.38; Fig. S3). Flux appears to be primarily driven by maximum water content at depth in soils (point colors in Fig. S3); however, observed surface methane flux generally increased as the ratio-scaled soil moisture increased. So, where the upper 1-m is wetter than average, fluxes are lower. Conversely, where the upper 1-m is drier than average, fluxes are higher. We attribute this finding to enhanced levels of methanogenesis in more saturated substrates at depth, where anaerobic conditions may be fueled by heightened levels of labile organic carbon and VWC (Figs. 4, S3), and to gas transport via preferential flow pathways through shallow and partially saturated soil conditions with enough water to limit oxidization. This empirical model was used to upscale local observations of methane fluxes across each ERT transect, where we mapped low/moderate/high methane flux classes (Fig. S3) onto the ERT-derived VWC transects (Fig. 4). Class thresholds of 0.5 and 0.75 VWC were selected based on groupings observed in the data (Fig. S3) as well as visual assessment of the spatial correlation with methane fluxes (Fig. 4). Flux levels are predicted to vary over small spatial scales (~1-5 m), and locations of high flux are predicted to occur with greater frequency lower on the hillslope. Areas of low predicted flux correlate with locations further upland and at the tops of mounds. Future refinements to our model may benefit from additional spatially-distributed data on factors (e.g., soil organic carbon, temperature, microbial communities) that could influence surface methane fluxes.

The ERT-derived VWC values were also found to oscillate spatially with an inverse correlation to topography (Fig. S4a,b). To quantify this periodic signal, we first averaged VWC

over two depth intervals (0-2 and 4-6 m). Then we de-meaned and bandpass filtered the VWC and topography records between 0.013-0.9 Hz corresponding to spatial periods of 2-75 m, effectively removing very high and low period signals, and computed the power spectrum of each record using a fast-fourier transform (FFT). The statistically significant spectral peaks were identified by fitting the data to a red noise exponential decay curve. We then used an F-test between the red noise level and the data to calculate the 90% confidence threshold, and retrieved statistically significant peaks with amplitudes greater than that level (Fig. S4c-h). The spatial period of spectral peaks represents the length (or distance) of one peak-trough cycle. Topography for both transects are dominated by a long period signal, between 63-75 m, and Line A also contains significant signals at shorter periods (e.g., 16 and 32 m) on par with thermokarst-mound sizes (<15 m diameter mounds with period cycles of <30 m; Fig. S4c,f). Water content at all depths and profiles also contain statistically significant periodic cycles consistent with thermokarst-mound sizes as well as longer period oscillations matching topography. Both ERT profiles also show shorter-distance oscillations in water content at shallow depths that disappear or lengthen at greater depths (Fig. S4d,e,g,h).

5.4 *Soil profiles of gas geochemistry and microbial analysis*

Gas content and the stable carbon isotope composition of methane ($\delta^{13}CH_4$) and carbon dioxide ($\delta^{13}CO_2$) were measured in multiple sediment depths of cores BH1 and BH6. The results show that both cores contained high amount of carbon dioxide (<12.3 mM CO_2) and methane (<3.9 mM CH_4) (Fig. 5). In core BH1, methane started to accumulate at around 120 cm depth with maximum concentrations of 3.1 mM around 200 cm. Carbon dioxide concentrations were highest in the upper ~1 m of soil, above the methane zone. The values of $\delta^{13}CH_4$ were around -60 ‰ in the upper methane zone and decreased to -70 ‰ at the main methane zone. Low $\delta^{13}CH_4$ values in the methane zone were accompanied by heavy $\delta^{13}C_{CO2}$ of -15 ‰ and high abundance of methanogenic archaea. Concurrently, qPCR analysis of the *mcrA* gene was consistent with these results. Elevated expression levels were found between 166-259 cm (peak at 166 cm, $5.3x10^6 \pm 3.5x10^5$ copies/1gr soil, Fig. 5a). In BH1 $\delta^{13}C_{CH4}$ reached ~-60‰ at 105 cm depth and $\delta^{13}C_{CO2}$ ~-28‰ at 65 cm depth, above the methane zone, coinciding with high methylobacter relative abundance at 56 and 68 cm depths (4% and 3.7% of total ASVs, respectively) and *pmoA* expression levels at 68 cm $4.05x10^7 \pm 1.02x10^6$ copies/1gr soil (Fig. 5a). The winter core, BH6, showed low concentrations of methane at the surface, with two peaks of methane accumulation. A first (higher) peak below 200 cm, and a second one below 500 cm (Fig. 5b). The relative abundance of methanogenic archaea and *mcrA* expression profile also supported in-situ methanogenesis at those depths, with a first peak between 198 cm 285 cm (0.2 % relative abundance of total ASVs and 28% of archaeal ASVs; $2.8x10^6 \pm 3.57x10^5$ copies/1gr soil). A second sharp peak was noted below 600 cm (0.7 % relative abundance of total ASVs and 28% of archaeal ASVs; $2x10^6 \pm 4.87x10^5$ copies/1gr soil) (Supplementary Data 3 and Fig. 5b).

The 16S amplicon-based sequencing revealed a diverse microbial community, with the abundance of almost all genera <1% (Supplementary Data 1). The taxonomic classification indicated the presence of at least 261 orders (262 bacteria and 9 archaea, Supplementary Data 2). Total relative abundance of bacterial ASVs was significantly higher than that of archaea (97.1% and 2.9%, respectively; Supplementary Data 1). Higher archaeal relative abundance was noted at 68 cm (6%) and 106 cm (6.1%) sediment depths of core BH1 and 655 cm (6.5%) and 711 cm (8.4%) of core BH6 (Supplementary Data 1). Among the bacteria, the prominent orders were Pseudomonadales (12.8%, Proteobacteria) and Micrococcales (11.6% of all ASVs,

Actinobacteria), followed by Burkholderiales (5.8%, Proteobacteria), Rhizobiales (Alphaproteobacteria, 4.6%) and Bacteroidales (4.3%, Bacteroidota). The predominant Archaean orders were Nitrososphaerales (1.2%) and Methanosarciniales (comprised of the genera *Candidatus* Methanoperedens, *Methanosarcina* and *Methanosaeta*), accounting for 0.8% of total ASVs (Supplementary Data 2). The genus *Pseudomonas* and family *Micrococcaceae* were the most prevalent classified ASVs (12.8% and 7.4%, of all ASVs, Supplementary Data 1).

The potential for in situ methanogenesis was assessed by relative abundance of methanogenic archaea. In core BH1, we identified methanogenic archaea of the family *Methanomassiliicoccaceae*, the genera *Methanosarcina*, *Methanoregula*, *Methanobacterium*, *Methanosphaerula*, *Methanosaeta* and Rice Cluster II. These were present at sediment depths between 106 cm to 259 cm of BH1 and were more prevalent at sediment depths of 166 cm and 186 cm, with relative abundances of 1.8% and 1.3%, respectively of total ASVs. Moreover, the overall relative abundance of methanogens in BH1 was 17.6% of archaeal ASVs and 0.3% of total ASVs (Supplementary Data 3). Toward the bottom of the 3.2-m summer core, the methanogens relative abundance significantly diminished; however, a fuller assessment would require analysis of the complete ~7m talik profile and quantification of active methanogens via RNA extractions.

Anaerobic methanotrophy in BH1 was suggested by the presence of *Candidatus* Methanoperedens, which was the predominant archaeon (6% at 68 cm and 5.5% at 106 cm of all ASVs, and 56% in all depths of archaeal ASVs; Supplementary Data 3). We also identified methanotrophic and methylotrophic bacteria in BH1 (relative abundance 2.4% of total ASVs, at sediment depths up to 106 cm and 1.6% throughout core BH1 of all ASVs; Supplementary Data 3). Of these, the predominant bacteria were the aerobic CH_4-oxidizing *Methylobacter* (59.6%) and *Methylotenera* (12.6%), which utilize methanol and methylamines oxidation, linked to denitrification[44,45]. The relative abundance of *Methylobacter* and *Methylotenera* (family *Methylophilaceae*) showed very similar patterns throughout the sediment depth profile of core BH1 (Supplementary Data 3), as expected[46,47]. Interactions between *Methylobacter* and *Methylotenera* have been previously reported. For example, transfer of nitrate-promoted methane-derived carbon[48] and of methanol[49]. However, in our study, spearman correlation between the two was not significant, probably due to the small sample size (n=20). In addition, we identified ASVs classified to the family level (*Methylophilaceae*), and may potentially represent the genus *methylotenera*.

The winter core BH6 included samples for microbial analysis up to a depth of 711 cm, enabling examination of complete talik profile. The relative abundance of the identified methanogens generally following the methane profile, overall accounting for 9.5% of archaeal ASVs and 0.2% of total ASVs (Fig. 5b, Supplementary Data 3). We identified methanogens of the family *Methanomassiliicoccaceae* and the genera *Methanosarcina*, *Methanoregula*, *Methanobacterium*, *Methanosphaerula*, as in BH1. In addition, methanogens of the order Methanomicrobiales and of the genus *Methanomassiliicoccus* were also noted (Supplementary Data 3). Unlike in the BH1 core, we did not identify methanogens of Rice Cluster II and the genus *Methanosaeta*. In contrast to the summer core BH1, *Candidatus* Methanoperedens was not identified at most depths in BH6. We only noted this methanotroph archaeon at low relative abundance between 162-198 cm (Supplementary Data 3).

In BH6, the only significant *pmoA* expression was identified at 100 cm depth ($1.3 \times 10^6 \pm 3.27 \times 10^5$ copies/1gr soil; Fig. 5b). Yet surprisingly, we observed high relative abundance of methanotrophic and methylotrophic bacteria (4%, Supplementary Data 3). These were more

abundant between 100-162 cm (4.9% and 7.7% of total ASVs, respectively) and between 655-711 cm (ranging between 2.7%-9.7% of total ASVs). While at shallower depths most ASVs were related to *Methylobacter* and *Methylotenera*, at deeper depths these were predominantly of the family *Methyloligellaceae* (Supplementary Data 3). Throughout BH6, these three accounted of the bulk of methanotrophic and methylotrophic bacteria (24.3%, 11.9% and 51.3%, respectively). Noteworthy, all three aforementioned bacteria can utilize methylated compound other than methane. The JS1 lineage (proposed Candidate phylum Atribacteria)[50] was abundant at sediment depths 166 cm and 186 cm of core BH1 (4.6% and 1.9% of total ASVs, respectively), together with the genus *Smithella* (Syntrophales; Desulfobacterota), with an abundance of 2.4% and 1.2% of total ASVs, respectively (Supplementary Data 1). JS1 bacteria have been reported in strictly anoxic organic-rich environments, gas hydrate-bearing sediments, and methanogenic environments[51]. Both JS1 bacteria and the *Smithella* genus have been suggested to perform syntrophic propionate and acetate oxidation in methanogenic environments[50,52-54], where propionate and acetate can be converted to methane by partner methanogens, contributing to methane emission as a greenhouse gas[53,54].

The relative difference in radiocarbon ages between pond bubbles and turf bubbles was higher for methane than carbon dioxide. Pond bubble $^{14}C_{CH4}$ (20,050 to 22,050 yrs BP) to was much older than the turf bubble $^{14}C_{CH4}$ (6,650 ±15 yrs BP). In contrast, $^{14}C_{CO2}$ ages were similar between pond (2,765 to 2,975 yrs BP) and turf (2,875 ± 20 yrs BP) bubbles. One explanation for these findings is that deeper thaw beneath ponds (Fig. S2) leads to more heavily ^{14}C-depleted permafrost organic matter substrates fueling methanogenesis; however, differences in methane and carbon dioxide production and gas transport pathways throughout the talik need to be more carefully studied.

Supplementary References

1. U.S. Geological Survey EROS Alaska Field Office, Alaska 300m digital elevation model (published 19970512), accessed April 22, 2010 at URL ftp://agdcftp.wr.usgs.gov/pub/dem/300m/akdem300m.tar.gz
2. Alaska Division of Geological and Geophysical Surveys. 2018. 3D Elevation Program 1.5-Meter Resolution Digital Terrain Model (published 20180316), accessed June 24, 2023 at URL https://elevation.alaska.gov/
3. Minsley, B. J., James, S. R., & Pastick, N. J. Alaska permafrost characterization: Geophysical and related field data collected in 2021. *U.S. Geological Survey Data Release*, doi.org/10.5066/P9XEMDE1 (2022).
4. James, S. R. *et al*. The biophysical role of water and ice within permafrost nearing collapse: Insights from novel geophysical observations. *JGR Earth Surface* **126**, doi.org/10.1029/2021JF006104 (2021).
5. Kass, M. A. *et al*. In situ nuclear magnetic resonance response of permafrost and active layer soil in boreal and tundra ecosystems. *The Cryosphere* **11**, 2943-2955 (2017).
6. Minsley, B. J., Pastick, N. J., Wylie, B. K., Brown, D. R., & Kass, A. M. Evidence for nonuniform permafrost degradation after fire in boreal landscapes. *Journal of Geophysical Research: Earth Surface* **121**, 320-335 (2016).
7. Minsley, B. J., Wellman, T. P., Walvoord, M. A., & Revil, A. Sensitivity of airborne geophysical data to sublacustrine and near-surface permafrost thaw. *The Cryosphere* **9**, 781-794 (2015).

8. Hoekstra, P. & McNeill, D. Electromagnetic probing of permafrost. Proc. *2nd Internat. Conf. On Permafrost. Nat. Acad. Sci*., 517–526 (1973).
9. Minsley, B. J. *et al.* Airborne electromagnetic imaging of discontinuous permafrost *Geophys. Res. Lett*. **39**, L02503 (2012).
10. Blanchy, G., Saneiyan, S., Boyd, J., McLachlan, P., & Binley, A. ResIPy, an intuitive open source software for complex geoelectrical inversion/modeling. *Computers & Geosciences* **137**, 104423. https://doi.org/10.1016/j.cageo.2020.104423 (2020).
11. Walsh, D. *et al.* A Small-Diameter NMR Logging Tool for Groundwater Investigations. *Groundwater* **51**: 914-926. doi.org/10.1111/gwat.12024 (2013).
12. Minsley, B. J. *et al*. Rapid and gradual permafrost thaw: A tale of two sites. *GRL* **49**, e2022GL100285, doi.org/10.1029/2022GL100285 (2022).
13. Walter Anthony, K. M. et al. A shift of thermokarst lakes from carbon sources to sinks during the Holocene epoch. *Nature* **511**, 452-456 (2014).
14. Elder, K. L., Roberts, M. L., Walther, T., & Xu, L. Single step Production of graphite from organic Samples for Radiocarbon Measurements. *Radiocarbon* **61**, 1843-1854. DOI: 10.1017/RDC.2019.136 (2019).
15. Stumm, W. and Morgan, J.J. Aquatic Chemistry, Chemical Equilibria and Rates in Natural Waters. 3rd Edition, John Wiley & Sons, Inc., New York (1996).
16. USGS PHREEQC Version 3. https://www.usgs.gov/software/phreeqc-version-3 (2021).
17. Bok, F., Moog, H. C., & Brendler, V. The Solubility of oxygen in water and saline solutions. *Front. Nucl. Eng*. **2**, doi.org/10.3389/fnuen.2023.1158109 (2023).
18. Hales, B. A. *et al.* Isolation and identification of methanogen-specific DNA from blanket bog peat by PCR amplification and sequence analysis. *Appl. Environ. Microbiol.* **62**, 668–675 (1996).
19. Bar-Or, I. *et al*. Iron-coupled anaerobic oxidation of methane performed by a mixed bacterial-archaeal community based on poorly reactive minerals. *Environ. Sci. Technol.* **51**, 12293–12301 (2017).
20. Holmes A. J., Costello A., Lidstrom M. E. & Murrell J. C. Evidence that particulate methane monooxygenase and ammonia monooxygenase may be evolutionarily related. *FEMS Microbiol. Lett.* **132**, 203–8. doi.10.1016/0378-1097(95)00311-R (1995).
21. Costello A. M. & Lidstrom M. E. Molecular characterization of functional and phylogenetic genes from natural populations of methanotrophs in lake sediments. *Appl. Environ. Microbiol.* **65**, 5066–74 (1999).
22. Mayr M. J. *et al*. Growth and rapid succession of methanotrophs effectively limit methane release during lake overturn. *Commun. Biol*. **3**, 108. (2020).
23. Caporaso, J. Ultra-high-throughput microbial community analysis on the Illumina HiSeq and MiSeq platforms. *ISME J* **6**, 1621–1624 https://doi.org/10.1038/ismej.2012.8 (2012).
24. Bolyen, E. *et al.* Reproducible, interactive, scalable and extensible microbiome data science using QIIME 2. *Nature Biotechnology* **37**, 852–857 doi.10.1038/s41587-019-0209-9 (2019).
25. Callahan, B. J. *et al.* DADA2: High-resolution sample inference from Illumina amplicon data. *Nature Methods* **13**, 581–583 doi: 10.1038/nmeth.3869 (2016).
26. Rognes, T., Flouri, T., Nichols, B., Quince, C. & Mahé, F. VSEARCH: a versatile open-source tool for metagenomics. *PeerJ* **4**, e2584 (2016).

27. Quast, C. *et al*. The SILVA ribosomal RNA gene database project: improved data processing and web-based tools. *Nucleic Acids Research* **41**, D590–D596. doi:10.1093/nar/gks1219 (2012).
28. Bokulich, N. A. *et al.* Optimizing taxonomic classification of marker-gene amplicon sequences with QIIME 2's q2-feature-classifier plugin. *Microbiome* **6**, doi.10.1186/s40168-018-0470-z (2018).
29. Pedregosa, F. *et al.* Scikit-learn: Machine Learning in Python. *Journal of Machine Learning Research* **12**, 2825–2830 (2011).
30. McMurdie, P. J., & Holmes, S. phyloseq: An R Package for Reproducible Interactive Analysis and Graphics of Microbiome Census Data. *PLoS ONE* **8**, e61217. doi.10.1371/journal.pone.0061217 (2013).
31. Wickham, H. ggplot2: Elegant Graphics for Data Analysis. Springer New York. Retrieved from https://zbmath.org/1397.62006 (2016).
32. Westermann, S., Langer, M., Boike, J., Heikenfeld, M., Peter, M., Etzelmüller, B., & Krinner, G. (2016). Simulating the thermal regime and thaw processes of ice-rich permafrost ground with the land-surface model CryoGrid 3. Geosci. Model Dev., 9(2), 523–546. https://doi.org/10.5194/gmd-9-523-2016
33. Nitzbon, J. *et al*. Pathways of ice-wedge degradation in polygonal tundra under different hydrological conditions. *Cryosphere* **13**, 1089–1123 (2019).
34. Nitzbon, J. et al. Fast response of cold ice-rich permafrost in northeast Siberia to a warming climate. *Nat. Commun.* **11**, 2201 (2020).
35. Whalen, S. C. & Reeburgh, W. S. Moisture and temperature sensitivity of CH4 oxidation in boreal soils. *Soil Biol. Biochem*. **28**, 1271–1281 (1996).
36. Oh, Y. et al. Reduced net methane emissions due to microbial methane oxidation in a warmer Arctic. *Nature Clim. Change* **10**, 317–321 (2020).
37. Mattson, M. D., & Likens, G. E. Air pressure and methane fluxes. *Nature* **347**, 718–719 (1990).
38. Fechner-Levy, E. J, & Hemond, H. F. Trapped methane volume and potential effects on methane ebullition in a northern peatland. *Limnol. Oceanogr*. **41**, 1375-1383 (1996).
39. Tokida, T. et al. Falling atmospheric pressure as a trigger for methane ebullition from peatland. *Glob. Biogeochem. Cycles* **21**, GB2003 (2007).
40. Xu, L., Lin, X., Amen, J., Welding, K. & McDermitt, D. Impact of changes in barometric pressure on landfill methane emissions. *Glob. Biogeochem. Cycles* **28**, 2013GB004571 (2014).
41. Czepiel, P. M. et al. The influence of atmospheric pressure on landfill emissions. *Waste Manag*. **23**, 593-598 (2003).
42. Forde, O. N. et al. Barometric-pumping controls fugitive gas emissions from a vadose zone natural gas release. *Sci Rep* **9**, 14080 (2019).
43. Varadharajan, C. & Hemond, H. F. Time-series analysis of high-resolution ebullition fluxes from a stratified, freshwater lake. *J. Geophys. Res*. **117**, G02004 (2012).
44. Kalyuhznaya, M. G. *et al.* Methylophilaceae link methanol oxidation to denitrification in freshwater lake sediment as suggested by stable isotope probing and pure culture analysis. *Environmental Microbiology Reports* **1**, 385-392 (2009).
45. Kalyuzhnaya, M. G. *et al.* Novel methylotrophic isolates from lake sediment, description of Methylotenera versatilis sp. nov. and emended description of the genus Methylotenera. *The ISME Journal* **62**, 106-111 (2012).

46. Beck, D. A. C. A metagenomic insight into freshwater methane-utilizing communities and evidence for cooperation between the Methylococcaceae and the Methylophilaceae. *Peer J* **1**, 1–23, doi.org/10.7717/peerj.23 (2013).
47. Oshkin, I. Y. *et al*. Methane-fed microbial microcosms show differential community dynamics and pinpoint taxa involved in communal response. *ISME Journal,* **9**, 1119–1129. doi.org/10.1038/ismej.2014.203 (2015).
48. van Grinsven, S., Sinninghe Damste, J. S., Harrison, J., Polerecky, L., & Villanueva, L. Nitrate promotes the transfer of methane-derived carbon from the methanotroph Methylobacter sp. to the methylotroph Methylotenera sp. in eutrophic lake water. *Limnol. Oceanogr.* **66**, 878-891 (2021).
49. Krause, S. M. *et al.* Lanthanide-dependent cross-feeding of methane-derived carbon is linked by microbial community interactions. *PNAS* **114**, 358-363 (2017).
50. Nobu, M. K. *et al.* Phylogeny and physiology of candidate phylum 'Atribacteria'(OP9/JS1) inferred from cultivation-independent genomics. *The ISME Journal* **10**, 273-286 (2016).
51. Lee, Y. M. *et al.* Genomic insight into the predominance of candidate phylum Atribacteria JS1 lineage in marine sediments. *Frontiers in Microbiology* **9**, 2909 (2018).
52. Leng, L. *et al.* A review on the bioenergetics of anaerobic microbial metabolism close to the thermodynamic limits and its implications for digestion applications. *Bioresource Technology* **247**, 1095-1106 (2018).
53. Nobu, M. K. Microbial dark matter ecogenomics reveals complex synergistic networks in a methanogenic bioreactor. *The ISME Journal* **9**, 1710-1722 (2015).
54. Westerholm, M., Calusinska, M., & Dolfing, J. Syntrophic propionate-oxidizing bacteria in methanogenic systems. *FEMS Microbiology Reviews* **46**, fuab057 (2022).
