# Supplementary material for: Upland Yedoma taliks are an unpredicted source of atmospheric methane
Source: Nat Commun. 2024 Jul 18;15:6056. doi: 10.1038/s41467-024-50346-5 (PMC11258132; doi:10.1038/s41467-024-50346-5)
Supplement: Supplementary file 3 — Description of Additional Supplementary Files [file 41467_2024_50346_MOESM3_ESM.pdf]

Description of Additional Supplementary Files

File Name: Supplementary Movie 1
Description: Subsurface cross-section of simulated thermokarst-mound and talik formation for the Lena river delta in northeastern Siberia under RCP8.5 and default parameter settings.

File Name: Supplementary Movie 2
Description: Subsurface cross-section of simulated thermokarst-mound and talik formation for the Lena river delta in northeastern Siberia under RCP8.5 and increased snowfall rate.

File Name: Supplementary Movie 3
Description: Subsurface cross-section of simulated thermokarst-mound and talik formation for the Lena river delta in northeastern Siberia under RCP8.5 and decreased snow density.

File Name: Supplementary Movie 4
Description: Subsurface cross-section of simulated thermokarst-mound and talik formation for the Lena river delta in northeastern Siberia under RCP8.5 and reduced lateral drainage efficiency.

File Name: Supplementary Movie 5
Description: Subsurface cross-section of simulated thermokarst-mound and talik formation for the Lena river delta in northeastern Siberia under RCP8.5 and modified mound geometry.

File Name: Supplementary Movie 6
Description: Subsurface cross-section of simulated thermokarst-mound and talik formation for the Lena river delta in northeastern Siberia under RCP8.5 and initial mound topography.

File Name: Supplementary Data 1
Description: 16S rRNA gene Genus table, obtained by next generation sequencing (NGS). ASVs were grouped at the Genus level. Full taxonomy is presented. Additional information provided is occurrence per sample, as absolute counts and relative abundance per Genus. Each sample is denoted by core number and sampling depth. Total read count and relative abundance of Bacteria, Archaea and total reads for each sample are presented.

File Name: Supplementary Data 2
Description: 16S rRNA gene Order table, obtained by next generation sequencing (NGS). ASVs were grouped at the Order level. Full taxonomy is presented. Additional information provided is occurrence per sample, as absolute counts and relative abundance per Order. Each sample is denoted by core number and sampling depth.

File Name: Supplementary Data 3
Description: 16S rRNA gene table of methanogens, methanotrophs and methylotrophs, obtained by next generation sequencing (NGS). Full taxonomy is presented. Additional information provided is occurrence per sample, as absolute counts. Each sample is denoted

by core number and sampling depth. Relative abundance is presented for the related groups by core and depth.
