# Supplementary material for: Upland Yedoma taliks are an unpredicted source of atmospheric methane
Source: Nat Commun. 2024 Jul 18;15:6056. doi: 10.1038/s41467-024-50346-5 (PMC11258132; doi:10.1038/s41467-024-50346-5)
Supplement: Supplementary file 11 — Reporting Summary [file 41467_2024_50346_MOESM11_ESM.pdf]

nature portfolio

Corresponding author(s): Katey Walter Anthony

Last updated by author(s): Jun 20, 2024

Reporting Summary

Nature Portfolio wishes to improve the reproducibility of the work that we publish. This form provides structure for consistency and transparency in reporting. For further information on Nature Portfolio policies, see our Editorial Policies and the Editorial Policy Checklist.

Statistics

For all statistical analyses, confirm that the following items are present in the figure legend, table legend, main text, or Methods section.

n/a	Confirmed	
☐	☒	The exact sample size (*n*) for each experimental group/condition, given as a discrete number and unit of measurement
☐	☒	A statement on whether measurements were taken from distinct samples or whether the same sample was measured repeatedly
☐	☒	The statistical test(s) used AND whether they are one- or two-sided<br>*Only common tests should be described solely by name; describe more complex techniques in the Methods section.*
☐	☒	A description of all covariates tested
☐	☒	A description of any assumptions or corrections, such as tests of normality and adjustment for multiple comparisons
☐	☒	A full description of the statistical parameters including central tendency (e.g. means) or other basic estimates (e.g. regression coefficient) AND variation (e.g. standard deviation) or associated estimates of uncertainty (e.g. confidence intervals)
☐	☒	For null hypothesis testing, the test statistic (e.g. *F*, *t*, *r*) with confidence intervals, effect sizes, degrees of freedom and *P* value noted<br>*Give P values as exact values whenever suitable.*
☒	☐	For Bayesian analysis, information on the choice of priors and Markov chain Monte Carlo settings
☒	☐	For hierarchical and complex designs, identification of the appropriate level for tests and full reporting of outcomes
☐	☒	Estimates of effect sizes (e.g. Cohen's *d*, Pearson's *r*), indicating how they were calculated

Our web collection on statistics for biologists contains articles on many of the points above.

Software and code

Policy information about availability of computer code

Data collection	Diffusive fluxes of methane and carbon dioxide were calculated using Python 3.11.5 code available through Elder and colleagues [2021, doi.10.1029/2020GB006922].<br><br>The datasets and scripts that were used for the preparation of Figure S5 b-c have been uploaded to the GitHub repository at: https://github.com/BergmanOded/Yedoma_Nat.Commun2024, digital resource identifier: https://zenodo.org/doi/10.5281/zenodo.11561649.<br><br>The model code and set-up files for the numerical modeling with CryoGrid are available from https://doi.org/10.5281/zenodo.3648266.
Data analysis	General eddy covariance data analysis, post-processing, gapfilling and NEE partitioning: Matlab R2017b, MS Excel 2016, and ReddyProc R package.<br>ReddyProc: Wutzler T, Lucas-Moffat A, Migliavacca M, Knauer J, Sickel K, Sigut, Menzer O & Reichstein M (2018) Basic and extensible post-processing of eddy covariance flux data with REddyProc. Biogeosciences, Copernicus, 15, doi: 10.5194/bg-15-5015-2018<br>Eddy covariance initial flux and biomet calculations: EddyPro 7.0.8<br>EddyPro: LI-COR Biosciences. (2021). Eddy Covariance Processing Software (Version 7.0.8) [Software]. Available at www.licor.com/EddyPro.<br>General geophysical data analysis: Python 3.8<br>Resistivity analysis software: ResIPy inversion software (version 3.3.2; Blanchy et al., 2020).<br>Blanchy, G., Saneiyan, S., Boyd, J., McLachlan, P., and Binley, A. (2020). ResIPy, an intuitive open source software for complex geoelectrical inversion/modeling. Computers and Geosciences, 137, 104423. https://doi.org/10.1016/j.cageo.2020.104423<br>NMR acquisition & processing software: Vista Clara acquisition (7.1.8) and processing (3.62/1.64) software (Vista Clara, Inc, Mukilteo, WA, USA)<br>Downstream microbial data analysis was performed in QIIME2 version 2020.11, classify-sklearn (version 0.23.1), and R version 4.3.2, using the

packages phyloseq, tidyverse, plyr, and ggplot2.
Statistical analyses: R version 4.2.2 packages, using car, ggplot2, MASS, and multcompView

For manuscripts utilizing custom algorithms or software that are central to the research but not yet described in published literature, software must be made available to editors and reviewers. We strongly encourage code deposition in a community repository (e.g. GitHub). See the Nature Portfolio guidelines for submitting code & software for further information.

Data

Policy information about availability of data

All manuscripts must include a data availability statement. This statement should provide the following information, where applicable:
- Accession codes, unique identifiers, or web links for publicly available datasets
- A description of any restrictions on data availability
- For clinical datasets or third party data, please ensure that the statement adheres to our policy

The raw sequence reads generated in this study have been deposited in the European Nucleotide Archive (ENA) at the EMBL European Bioinformatics Institute (EMBL-EBI) Database (https://www.ebi.ac.uk/ena/browser/home) as BioProject accession number PRJEB59938 (https://www.ebi.ac.uk/ena/browser/view/PRJEB59938). Eddy covariance data generated in this study have been deposited at AmeriFlux and are available as site US-YNS (North Star Yedoma) under accession code https://ameriflux.lbl.gov/sites/siteinfo/US-YNS. Chamber-based methane flux data generated in this study have been deposited in the Arctic Data Center under accession code https://doi.org/10.18739/A26W96B49. Geophysical data generated in this study have been deposited in the U.S. Geological Survey database under accession code https://doi.org/10.5066/P9XEMDE1. Source data are provided with this paper. Additional data are available in the Supplementary Information and Supplementary Data sections.

Research involving human participants, their data, or biological material

Policy information about studies with human participants or human data. See also policy information about sex, gender (identity/presentation), and sexual orientation and race, ethnicity and racism.

Reporting on sex and gender	n/a
Reporting on race, ethnicity, or other socially relevant groupings	n/a
Population characteristics	n/a
Recruitment	n/a
Ethics oversight	n/a

Note that full information on the approval of the study protocol must also be provided in the manuscript.

Field-specific reporting

Please select the one below that is the best fit for your research. If you are not sure, read the appropriate sections before making your selection.

☐ Life sciences ☐ Behavioural & social sciences ☒ Ecological, evolutionary & environmental sciences

For a reference copy of the document with all sections, see nature.com/documents/nr-reporting-summary-flat.pdf

Ecological, evolutionary & environmental sciences study design

All studies must disclose on these points even when the disclosure is negative.

Study description	This study included eddy covariance measurements of methane (CH4) and carbon dioxide (CO2) fluxes at North Star Yedoma (NSY), a grassland field in interior Alaska characterized by thermokarst (thaw) mounds forming due to degradation of ice-rich yedoma, polygonal-ground permafrost soil. Plot-scale measurements of the same gas fluxes were made using a portable chamber system at NSY and 25 other extensive thermokarst-mound study sites in Alaska and adjacent controls (lacking thermokarst mounds). Microtopographical position, soil temperature and moisture were recorded for most observations. At NSY we made geophysical measurements of soil properties and conducted borehole soil analyses. Soil analyses included determination of dry density; organic carbon and nitrogen concentrations; soil dissolved CH4 and CO2 concentrations; δ13C values of CH4 and CO2; qPCR analysis of the mcrA and pmoA genes, and 16S amplicon-based sequencing for characterization of the microbial community. We used remote-sensing detection of thermokarst-mound occurrences in the pan-Arctic, and numerical modeling of talik development in Northern Siberia.
Research sample	A sample for the eddy covariance tower data was a 30-min average of 10hz observation of carbon fluxes and 1Hz observation of

Research sample

meteorological data at NSY expressed as µmol m-2 s-1; each observation was scaled up to daily rates (mg m-2 d-1). A sample for plot-scale chamber fluxes was an observation (typically five minutes) at thermokarst-mound and control (lacking thermokarst mounds) study sites. A sample for borehole soil core analysis was a 3 to 100 cc subsample from individual cores. These samples are intended to represent the populations of the NSY study site fluxes (EC tower samples); thermokarst-mound study site fluxes (chambers) and vertical soil profiles at mid-field elevation at NSY (borehole samples). Our study also involved an existing dataset of seasonal and annual methane fluxes from other northern sites (Treat, C. C., Bloom, A. A., & Marushchak, M. E. Nongrowing season methane emissions – a significant component of annual emissions across northern ecosystems. Global Change Biology 24, 3331-3343 (2018).)

Sampling strategy

The eddy covariance tower was located in the center of the grassland field to maximize representation of the thermokarst grassland in the footprint. Plot-scale measurements at NSY and other thermokarst-mound study sites were performed at regular intervals along transects oriented so as to capture both macrotopographical gradients (high to low field-scale elevations) and microtopographical variability (thermokarst mound tops, flanks, and trenches). For the primary comparison of fluxes between NSY and other extensive thermokarst mound sites, we used large sample sizes (n=665 NSY, n=514 other). Since comparison to non-thermokarst sites (controls) was made primarily to the literature, our sample size for control site measured fluxes was smaller (n=78). Two borehole soil cores located less 60 cm apart were subsampled in winter and summer for physical and biogeochemical analyses at <20 cm intervals. Soil subsamples for microbial analyses were selected from a smaller subset of dispersed depths within the cores. Sample size-predetermination calculations were not performed; however, we followed standard sample size collection procedures in our field (Treat et al. 2018 https://doi.org/10.1111/gcb.14137; Euskirchen et al. 2020 doi.org/10.1007/s10021-019-00460-z; Carnevali et al. 10.3389/fmicb.2018.01192).

Data collection

Colin Edgar was responsible for eddy covariance data collection (net ecosystem exchange of CO2 and CH4 flux and meteorological data). These data were collected from May 8, 2021 to May 14, 2023. The eddy covariance system was mounted at 2 m height on a tripod in the center of the NSY grassland study field (64.894 °N, 147.637 °W). Footprint analyses indicate that at least 80% of the field-scale flux contribution came from the grassland area within the target thermokarst-mound ecosystem. The remainder included a portion of the reforested thermokarst mounds. Within the 80% flux contribution area, thermokarst ponds and their associated littoral vegetation comprised a maximum of 2.4% of the area and 7.9% (0.8%) of the CH4 source observed by the EC tower in summer (winter). Until November 2022, when line power was established at NSY, electrical power for instrumentation was provided by a single 250W solar panel and twelve 6-volt absorbent glass mat batteries, with the weekly use of a generator for ~12-hour battery charging during the months of December and January. Flux data collected during generator run time were discarded. The eddy covariance instrumentation included a 3-D sonic anemometer (CSAT-3B; Campbell Scientific Instruments, Logan, Utah, USA), an open-path infrared gas analyzer for CO2¬, water, and energy fluxes (LI-7500DS IRGA; LI-COR, Lincoln, Nebraska, USA), and a fast-response open-path CH4 analyzer (LI-7700; LI-COR, Lincoln, Nebraska, USA). The main axis of the LI-7500DS IRGA was tilted by 30° with respect to the horizontal to aid in draining condensation and precipitation from the optical windows. The sonic anemometer and both IRGAs were mounted on a shared horizontal bar and were laterally separated by <20 cm to reduce flux loss and flow distortion. All fast response instrumentation was connected to a SmartFlux3 (SF) unit (LI-COR, Lincoln, Nebraska, USA) to log raw data at 10 Hz. Raw 10Hz and 30min mean data were stored on the SF USB drive. The gas analyzers were calibrated every 3-4 months following the instructions in the instrument manuals [LI-COR Inc., 2020] since inspections indicated that the instruments remained stable over that time frame. Basic microclimatic data were also collected, including air temperature (Ta) and relative humidity (RH; 2m above the ground; EE181, Campbell Scientific Instruments), soil water content (VWC; at 15cm depth, CS616, Campbell Scientific Instruments), precipitation (P; at 0.2 m; TE525MM, Texas Electronics, Dallas, Texas, USA), short and longwave radiation components, net radiation and albedo (SWin/out, LWin/out Rn, albedo; at 2m above the ground; NR-01; Hukseflux, Delft, Netherlands), soil temperature (Ts at 1 and 10 cm depth; 107 thermistor probe; Campbell Scientific Instruments), and barometric pressure (Pa; Licor LI-7500DS). These variables were measured at 1Hz and stored on the datalogging system (CR1000X; Campbell Scientific Instruments). Both the processed eddy covariance and microclimatic data were averaged for 30 min periods. Raw flux data acquisition during the entire period was about 67% after accounting for data loss from power outages, instrument malfunction, precipitation, and initial flux processing filtering. Data post-processing such as u* filtering and quality flagging further reduced data coverage to about 54%. Data gaps occurred because of instrument malfunction, power outages, or occasional generator use in December and January. Shorter gaps in the eddy covariance data were usually related to instrument errors during precipitation events in the summer and winter. Longer gaps occurred due to power outages and instrument shutdown during cold temperatures. For data gaps in net ecosystem exchange (NEE) and CH4 of approximately 1–6 days, we gap filled by calculating the mean diurnal variation, where a missing observation is replaced by the mean for that time period (half hour) based on adjacent days. This method provided stable approximations of missing data using 7-day independent windows during the nighttime hours and 14-day windows during the daytime hours.
Katey Walter Anthony and Nicholas Hasson collected plot-scale (660 cm2) chamber flux data on a seasonal basis from March 18, 2020 through September 19, 2023 using portable chambers following methods described in Elder and colleagues [2021, doi.10.1029/2020GB006922]. Chambers consisted of opaque, plastic 20-L buckets with bottoms removed and resealable air-tight lids (Gamma Seal Lid, Encore Plastics, Sandusky, OH, USA). Chambers blocked 88% (41%) of photosynthetically active radiation measured with a cosign quantum flux sensor (Apogee Instruments, Logan, UT) in full sun (shade). Chambers were placed on the ground surface, snow surface, or on semi-permanent collars for individual flux measurements. Air was recirculated through the chamber and either a Los Gatos Research Ultra-Portable Greenhouse Gas Analyzer (UGGA) or Los Gatos Research Micro-Portable Greenhouse Gas Analyzer (MGGA) (ABB INC., 85 Quebec City, CA) with a CH4 concentration measurement frequency of 1 Hz. Diffusive fluxes were calculated from the ideal gas law using chamber volume, temperature, atmospheric pressure measured via the eddy covariance tower's LI-COR LI7700 (LI-COR Inc., Lincoln, Nebraska, USA) operating mid-field at NSY. The slope of linear CH4 concentration change (usually R2 > 0.90 correlation to linear least squares fit) for a minimum of 45 seconds (45 observations) and a maximum of 210 seconds (45 < n > 210) was used to determine mass change within the chambers. Observations of zero (neutral) flux were also included based on no change in concentration within chambers. Observations with non-linear concentration change (R2 of linear fit < 0.80), no linear sections more than 45 seconds, or with stepwise concentration increases (interpreted as ebullition) were omitted to ensure that the reported measurements represent purely diffusive fluxes. These strict protocols eliminated any disturbance cause by placing the chamber, which we assume would manifest as non-linear CH4 concentration change. See Elder and colleagues [2021, doi.10.1029/2020GB006922] for more details regarding the processing of raw chamber data and data quality protocols.
Alexander Kholodov and Nicholas Hanson extracted soil cores. Katey Walter Anthony performed the soil core subsampling in the field. Katey Walter Anthony, Efrat Eliani-Russak and Oded Bergman performed laboratory measurements of soil core subsamples. We sampled NSY soils in summer and winter by drilling boreholes, BH1 and BH6, on September 15, 2021 and March 18, 2023, respectively. These boreholes, 60-cm apart, were located on top of a thermokarst mound at mid elevation in the NSY study field adjacent to the eddy covariance tower. BH1 was drilled with a gas-powered AMS frozen soil auger with 5 cm of internal (core) diameter (https://www.ams-samplers.com/frozen-soil-auger-kit/). Except for the porewater gas analyses, soil subsampling at 10 to

15-cm intervals along the 3.2-m BH1 core was not volumetrically controlled. Soil samples were placed in ziploc bags, and transferred to the freezer. In winter we collected the BH6 core using a Talon Drill system, which enabled us quantify soil dry density in 56 samples in volumetrically-controlled samples distributed throughout the ~7.0 m deep talik and underlying permafrost soil down to 7.25 m. In the lab, we determined gravimetric soil moisture content as weight loss dried at 105 °C expressed as percentage of wet weight. We calculated volumetric moisture content of talik soils as the product of gravimetric moisture content and dry density. Since dry density was not measured on BH1, we used a depth-dependent dry density relationship determined from BH6. Percent water saturation, which is the ratio of the total volume of voids to the volume of free water in a sample, was calculated as the product of water content and specific gravity divided by the void ratio. We adjusted the specific gravity of silt with low organics (2.65) to account for organic matter concentration, calculated as 1.8 times the measured organic carbon concentration. To determine dissolved CH4 and CO2 concentrations and the stable carbon isotopic composition (δ13C) of CH4 and CO2, a 3 ml soil plug was collected with a tip-cut 5 ml syringe from each depth section of the BH1 and BH6 cores and placed in a 20 ml glass serum containing preboiled and cooled 5M NaCl solution (inhibiting microbial activity). The vials were closed immediately with butyl rubber stoppers (GMT) and aluminum crimp caps with no headspace (this was achieved by inserting a needle into the stopper, while it was closed, allowing some of the salt water out, making some room for the relatively massive stopper), and then stored upside down until processing. In the lab, a 3 mL gas headspace at atmospheric pressure was created in each porewater sample vial by addition of ultrapure nitrogen using two sterile syringes. Samples were vigorously shaken for two minutes and analyzed for CH4 concentration. After two weeks equilibration at 22 ºC, we measured the CO2 and O2 concentration in samples. Porewater dissolved CH4, CO2 and O2 concentrations were calculated according to the ideal gas law, considering Henry's constants (KH) in the salt solution. Methane concentration was analyzed on a Focus Gas Chromatograph (GC) system (Thermoscientific, Germany) equipped with a flame ionization detector and shinCarbon ST packed column (Restek, USA). Injection volume was 240 µL. O2 concentration was measured by injecting 1 mL of diluted headspace into a 10 mL serum vial crimped with blue silicon septa, flushed with ultrapure helium. The vial was fitted with a PSt6 sensor spot that was monitored by Fibox3 (Presens, Germany), capable of measuring O2 at ppb levels (1% O2 mix was used for calibration of the sensor spot). The δ13C of CH4 and CO2 values and CO2 concentrations were determined using Gas Source Isotopic Ratio Mass Spectrometer (GS-IRMS) interfaced with a preconcentrating device (PreCon) and Gas Bench II (Thermo Scientific, Germany), equipped with 1 mL sample loop and Pora plot GC (Rt-Q-Bond, fused silica PLOT Restek, USA) for CO2 separation. Samples of 100 to 1000 µL were injected into ultrapure helium flushed vials (12 mL, Labco UK) to be measured. All gas standards had been calibrated against reference materials from NIST 8559, 8560, and 8561 (for CH4) or NBS-19 and NBS-18 (for CO2), using the GS-IRMS, or were provided from Air Gas (Air Liquide, Plumsteadville, PA, USA) or from Isometric Instruments (GASCo, Victoria, BC, Canada) with certificates. Isotope values are expressed in units of per mill (‰) in the typical δ-notation vs. V-PDB. DNA extraction (approximately 0.25 gr) was performed on subsamples of the summer and winter cores using the PowerSoil™ DNA Isolation Kit (QIAGEN, Hilden, Germany; prior MoBio, CA, USA), according to the manufacturer's instructions. DNA extracts were subsequently stored at -80 °C. The gene expression of mcrA and pmoA, was assessed from the DNA extractions by qPCR, utilizing primer sets ME1F (forward, 5'-GCMATGCARATHGGWATGTC-3') and ME3R (reverse, 5'-TGTGTGAASCCKACDCCACC-3') for the mcrA gene and A189gc (forward, 5'-GGNGACTGGGACTTCTGG-3') and mb661 (reverse, 5'-CCGGMGCAACGTCYTTACC-3') for the pmoA gene. qPCR reactions were run in triplicates, each, in a total volum of 20 µl, containing 10 µl Fast SYBR™ Green Master Mix (Applied Biosystems™). Forward and reverse primer concentrations were 0.25 mM for the mcrA gene and 0.5 mM for the pmoA. Reactions were performed using Rotor Gene 6000, software version 1.7 (Corbett Research, UK), with the following conditions: 20 sec activation at 95 °C, followed by 40 cycles of denaturation of 95 °C for 3 sec, annealing of 53 °C (mcrA) and 56 °C (pmoA) for 30 sec and an extension step of 30 sec at 72 °C. gBlocks dsDNA fragments were used as standard calibration curves for mcrA (Methanolacinia petrolearia DSM 11571; GenBank accession number: NC_014507.1) and pmoA (Methylococcus capsulatus particulate methane monooxygenase 27 kDa subunit (pmoA); GenBank accession number: L40804.2) (Integrated DNA Technologies, Coralville, IA, USA). Standard calibration curves with known concentration of the target gene (gBlocks) vs cycle threshold (CT) were used to determine gene concentration. 16S gene amplicon-based sequencing (targeting the V4 region), was performed using the modified primer pair with consensus sequences CS1_515F (ACACTGACGACATGGTTCTACAGTGCCAGCMGCCGCGGTAA) and CS2_806R (TACGGTAGCAGAGACTTGGTCTGGACTACHVGGGTWTCTAAT) (Sigma-Aldridge, Israel) [105]. The first PCR was performed in 25 µl reactions, containing 12.5 µl KAPA HiFi HotStart ReadyMix (KAPA Biosystems, Wilmington, WA, USA), 0.75 µl forward and reverse primers at a final concentration of 300 nM each. PCR conditions were: an initial denaturation at 95 °C for 3 min, followed by 30 cycles of 98 °C for 20 sec, 60 °C for 15 sec and 72 °C for 30 sec. PCR products were inspected on a 2% agarose gel to measure bands relative intensity. Samples were pooled and purified using calibrated Ampure XP beads and used for library preparation. PCR visualization, purification, library preparation and sequencing (2 × 250 bp pair-end reads) were performed at HyLabs (Israel), and sequenced on an Illumina MiSeq platform. Demultiplexing of the paired end reads and subsequent analysis was done using QIIME2 V.2020-11. Quality of sequenced reads was assessed using the q2-demux plugin, followed by chimera detection, and merging of reads into Amplicon Sequence Variants (ASVs) with DADA2, using the q2-dada2 plugin. To account for length variations, ASVs were defined by clustering at 100% similarity. Taxonomy was assigned using the QIIME-release of the 138-SILVA database (99% clustering). Extract-reads and fit-classifier-naive-bayes methods were used for the classifier, via the q2-feature-classifier plugin and classification of the ASVs by the classify-sklearn method (ver. 0.23.1).
Burke Minsley, Stephanie James and Neal Pastick were responsible for geophysical data. Ground-based ERT data were collected at NSY along two near-perpendicular transects using an AGI SuperSting R8 system (Advanced Geosciences, Inc., Austin, Texas) on September 15, 2021. Survey lines comprised 112 stainless steel electrodes at 2 m spacing for a total length of 222 m. The North Star A transect ran from northeast to southwest across the cleared field, starting next to a small pond at the edge of the field and ending in the forest where a large trench was crossed. The North Star D profile ran downslope, starting in the forest at the south edge of the field and ending just north of the intersection with the North Star A transect. The locations and orientations of the ERT transects were chosen to maximize overlap with chamber measurement locations. Electrical resistivity data were acquired using a dipole-dipole array, and data were inverted using ResIPy inversion software to produce 2-D profiles of resistivity along each transect. Results are shaded with a white overlay at depth in areas of low data sensitivity where model parameter uncertainty is greater. Borehole NMR data were collected in BH1 between 0.25 – 2.25 m depths, with 0.25 m vertical sampling intervals using a Dart in situ soil moisture probe (Vista Clara, Inc., Mukilteo, Washington). NMR data were analyzed using proprietary software (Vista Clara, Inc.) to produce estimates of total water content and pore size distribution based on multiexponential fitting to the measured T2 decay curve data. Total volumetric liquid water content for the mobile, capillary, and bound components of the pore space was derived from the integrated partial water contents (water content as a function of T2 decay time constant, computed from the multiexponential fits) with time constant values less than 3.16 ms, between 3.16 ms and 31.6 ms, and greater than 31.6 ms, respectively.

Timing and spatial scale

Eddy covariance data for the single NSY grassland field were collected once for two years, from May 8, 2021 through May 14, 2023. Plot-scale (660 cm2) chamber fluxes were collected seasonally (summer, winter and spring) from March 2020 through September

2023 among 26 Alaska thermokarst mound sites. Borehole soil cores were collected twice near the NSY eddy covariance tower on September 15, 2021 and March 18, 2023. Geophysical data were collected once along two field-scale transects at NSY on September 15, 2021.

Data exclusions

Eddy covariance tower flux data collected during generator run time in winter were discarded.
Among chamber flux measurements, observations with non-linear concentration change ($R2$ of linear fit < 0.80), no linear sections more than 45 seconds, or with stepwise concentration increases (interpreted as ebullition) were omitted to ensure that the reported measurements represent purely diffusive fluxes.

Reproducibility

No lab experiments were conducted in this study. We verified reproducibility of field observations by repeating measurements at multiple sites, seasons and years.

Randomization

Flux observations were assigned to primary groups according to field site type: NSY, other thermokarst-mound sites, and control sites (lacking thermokarst mounds). Within groups, observation plots followed stratified random sampling along macrotopographical and microtopographical field gradients. The NSY study site was chosen for eddy covariance flux monitoring and other intensive measurements because it is a large open field that meets the requirements of eddy-flux; has electrical grid access; and has thermokarst features, vegetation and hydrology similar to those of our other extensive study sites where thaw initiated in the last 40 to 70 years. The other extensive study sites were selected based on the occurrence of thermokarst mounds (mostly observed a priori in Lidar imagery), reasonable access from road systems, property ownership permissions.

Blinding

A priori selection of field study sites using Lidar to identify thermokarst-mound presence was a blinding process because no other information about vegetation, soil moisture or carbon fluxes was available to bias our site selection decision. In the field, chamber placement on terrestrial land surfaces was also a blind process in that locations of hotspot methane fluxes were not obvious to field workers, which is not the case in thermokarst lakes, where bubble plumes indicating methane hotspots can be seen.

Did the study involve field work? ☒ Yes ☐ No

Field work, collection and transport

Field conditions

Field work was conducted in a variety of conditions, mostly between dawn and dusk. Rain/snow precipitation and strong wind during field work were rare. In winter, researchers navigated through snow and took precautions to insulate equipment against freezing temperatures.

Location

The NSY study site is located seven kilometers NW of Fairbanks, Alaska in interior Alaska (64.8939 °N, 147.6373 °W). NSY is part of a ~60-hectare field of thermokarst mounds formed following anthropogenic disturbance to the mature black spruce forest which occurred sometime between August 1976 and September 1978. The talik (thaw bulb) depth ranges from 5 to 9 meters. From 1970 to 2022 this subarctic, continental region had a mean annual air temperature of −2.2 ± 1.2°C and mean annual precipitation of 288 ± 75 mm water equivalent. The NSY thermokarst-mound field is dominated by two native grasses, Calamagrostis lapponica and Calamogrostis canadensis. Twenty-two other interior Alaska thermokarst-mound study sites are located within 30 km of NSY and vary according to time since disturbance and present-day vegetation cover (deciduous forest, coniferous forest, grassland). One thermokarst-mound site is located near Hess Creek in the boreal forest (65.5620 °N, 148.9142 °W). Two thermokarst mound sites are located in northern Alaska in tundra near Toolik Field Station (68.6461 °N, 149.4450 °W).

Access & import/export

Research at NSY was conducted with permission of property owner, Roger and Melinda Evens beginning in March 2020. In August 2021, new property owner, Midnight Sun Golf Course – Nadon Family Home, LLC granted permission for continued research and a formal land use permit to the University of Alaska Fairbanks was granted Feb. 3, 2023. Borehole soil samples (geologic samples) were collected at NSY and subsamples exported to Israel in a responsible manner and in accordance with relevant permits and local laws. At our other extensive study sites, we obtained property owner permission to measure short-term chamber fluxes, soil temperature and moisture without leaving any trace, and no samples were removed from the extensive sites as part of this research.

Disturbance

Field sites were accessed on foot with backpacks and sleds, minimizing disturbance. Borehole drilling was a light-weight activity with no disturbance to NSY other than the boreholes themselves, which were subsequently instrumented with loggers and soils replaced. Soil plugs, removed with spades at a subset of sites to measure consecutively deeper fluxes, were restored to their original positions after the measurements with negligible disturbance to the soils/vegetation.

Reporting for specific materials, systems and methods

We require information from authors about some types of materials, experimental systems and methods used in many studies. Here, indicate whether each material, system or method listed is relevant to your study. If you are not sure if a list item applies to your research, read the appropriate section before selecting a response.

Materials & experimental systems

n/a	Involved in the study
☒	☐ Antibodies
☒	☐ Eukaryotic cell lines
☒	☐ Palaeontology and archaeology
☒	☐ Animals and other organisms
☒	☐ Clinical data
☒	☐ Dual use research of concern
☒	☐ Plants

Methods

n/a	Involved in the study
☒	☐ ChIP-seq
☒	☐ Flow cytometry
☒	☐ MRI-based neuroimaging

Plants

Seed stocks	n/a
Novel plant genotypes	n/a
Authentication	n/a
